# GATA4/FOG2 transcriptional complex regulates *Lhx9 *gene expression in murine heart development

**DOI:** 10.1186/1471-213X-8-67

**Published:** 2008-06-24

**Authors:** Fatima O Smagulova, Nikolay L Manuylov, Lyndsay L Leach, Sergei G Tevosian

**Affiliations:** 1 Department of Genetics, Dartmouth Medical School, Hanover, NH 03755, USA; 2 Norris Cotton Cancer Center, Dartmouth Medical School, Hanover, NH 03755, USA; 3 Institute of Chemical Biology and Fundamental Medicine, Russian Academy of Sciences, Novosibirsk, Russia

## Abstract

**Background:**

GATA4 and FOG2 proteins are required for normal cardiac development in mice. It has been proposed that GATA4/FOG2 transcription complex exercises its function through gene activation as well as repression; however, targets of GATA4/FOG2 action in the heart remain elusive.

**Results:**

Here we report identification of the *Lhx9 *gene as a direct target of the GATA4/FOG2 complex. We demonstrate that the developing mouse heart normally expresses truncated isoforms of *Lhx9 *– *Lhx9α *and *Lhx9β*, and not the *Lhx9-HD *isoform that encodes a protein with an intact homeodomain. At E9.5 *Lhx9α/β *expression is prominent in the epicardial primordium, septum transversum while *Lhx9-HD *is absent from this tissue; in the E11.5 heart LHX9α/β-positive cells are restricted to the epicardial mesothelium. Thereafter in the control hearts *Lhx9α/β *epicardial expression is promptly down-regulated; in contrast, mouse mutants with *Fog2 *gene loss fail to repress *Lhx9α/β *expression. Chromatin immunoprecipitation from the E11.5 hearts demonstrated that *Lhx9 *is a direct target for GATA4 and FOG2. In transient transfection studies the expression driven by the cis-regulatory regions of *Lhx9 *was repressed by FOG2 in the presence of intact GATA4, but not the GATA4^ki ^mutant that is impaired in its ability to bind FOG2.

**Conclusion:**

In summary, the *Lhx9 *gene represents the first direct target of the GATA4/FOG2 repressor complex in cardiac development.

## Background

Unlike many developing organs that remain dormant until the time of birth, a properly functioning embryonic heart is essential for embryo survival. Hence, defects in cardiac function are a common cause for embryonic lethality. Gene targeting in mice revealed multiple genes that are required for cardiac development and function; however, the interplay between these genes often remains a mystery. This is especially true for genes encoding for transcription factors which are expressed in a dynamic fashion in various cellular compartments that constitute the developing heart.

Friend of GATA, member 2 gene (FOG2, ZFPM2 – Mouse Genome Informatics) is prominently expressed in multiple cell types that constitute the embryonic heart [1]. To examine the function for FOG2 in cardiac development gene-targeted mice have been generated [2]. *Fog2*^-/- ^(null) embryos die at mid-gestation (~E13.5) with a cardiac defect characterized by an atrial septal defect, thin ventricular myocardium, common atrioventricular (AV) canal and the Tetralogy of Fallot malformation. Of particular additional interest is the finding that the development of cardiac vasculature was blocked in *Fog2*^-/- ^mice. Despite the apparently normal formation of an intact epicardial layer and expression of epicardium-specific genes in *Fog2 *null mutants, markers of cardiac vessel development (ICAM-2 and KDR) are not detected, indicative of failure to activate their expression and/or to initiate the epithelial to mesenchymal transformation of epicardial cells [2,3]. These results are particularly insightful with respect to KDR, since KDR (FLK1, VEGFR2), the major receptor for VEGF (vascular endothelial growth factor), is an important marker of vascular cells and is absolutely essential for vascular development (e.g. [4], see [5] for a review).

Although gene targeting revealed the requirement for FOG2 in multiple aspects of cardiac development, the specific genetic program (or programs) downstream of FOG2 remained unknown. Research by us and others established that multi-type zinc-finger proteins of the FOG family (FOG1, FOG2, xFOG and dUSH) control biological activities of GATA transcription factors (for review, see [6,7]). Based on the results from the hematopoietic system it has been suggested that FOG proteins serve as co-factors for GATA family members by forming a GATA/FOG complex on specific GATA sites. Indeed, differentially regulated genes have been identified for GATA1-FOG1 complex vs. GATA1 alone in blood development (e.g. [8]. Furthermore, these studies demonstrated that FOG1 could stimulate or inhibit GATA1 activity depending on cell and promoter context [9]. For example, FOG1 stimulates GATA1 activity on the p45 NF-E2 gene promoter, which is active in erythroid cells and megakaryocytes [10]; however, it represses GATA1 activity on the erythroid-specific *Eklf *and transferrin receptor II promoters, as well as on a synthetic GATA1-dependent promoter [11,12]

Earlier studies aimed at the similar characterization of the GATA4-FOG2 relationship with respect to cardiac development relied on transient transfection assays with well-characterized *Anf*, *Bnp *and *α-Mhc *promoters that contain GATA sites [13,14]. However, these genes are normally expressed in mutant *Fog2 *null or *Gata4*^*ki/ki *^hearts (the V217G *Gata4 *mutation [15] which specifically cripples the interaction between GATA4 and FOG proteins) arguing against the essential role for GATA4/FOG2 complex in their regulation [2,15,16]. Hence, transcriptional targets of GATA4/FOG2 complex in the heart are currently unknown.

The cardiac *Gata4*^*ki/ki *^phenotype showed numerous similarities to the *Fog2 *null heart underscoring the role for GATA4/FOG2 protein complex in cardiac gene regulation [15]. However, the mode of GATA4/FOG2 action, as a repressor or activator, remains unclear and necessitates identification of its downstream targets. In order to identify the targets of GATA4/FOG2 action in the mammalian heart we performed several Affymetrix microarray comparisons of gene expression in normal and mutant E13.5 hearts. As many groups have identified the regions in FOG proteins that mediate their function as GATA co-repressors [17-22], we expected that gene up-regulation (de-repression) should be, at least partially, responsible for causing cardiac syndrome in the GATA4/FOG2 mutants.

Here we describe one of the targets of GATA4/FOG2 complex in its transcriptional repressor role, *Lhx9 *gene. LHX9 belongs to the family of the LIM-HD (LIM-homeodomain) proteins. The roles for the majority of LIM-HD encoding genes have been mostly defined in the context of nervous system development where they act to specify neuronal identities of post-mitotic neurons (reviewed in [23]). *LIM-HD *genes act in a context-dependent fashion, cooperating with other factors to establish enormous diversity of the nervous system [24]. The function for LHX9 was proposed in the development of the nervous system as this gene is prominently expressed in the motoneurons of the spinal cord and in the developing brain [25-28]. Despite this prominent neuronal expression pattern, knockout of *Lhx9 *in mice did not affect animal viability or neuronal development probably reflecting a redundancy of *Lhx9 *and its close structural relative, *Lhx2 *[29]; unexpectedly, the knockout revealed a requirement for *Lxh9 *in early gonad formation [29]. Our studies demonstrate that GATA4/FOG2 transcription complex regulates *Lhx9 *cardiac expression and suggest that the role for *Lhx9 *in the development of the heart should be evaluated.

## Results

### Cardiac expression of the *Lhx9* gene splicing isoforms

In order to identify the targets of GATA4/FOG2 action in mammalian heart development we performed an Affymetrix microarray comparison of gene expression in normal and *Fog2 *null E13.5 hearts. The microarray profiling yielded surprisingly few genes that were differentially (~2.5 times up- or down-regulated) expressed in the mutant samples vs. controls (Additional File 1 and data not shown). Importantly, the probe set corresponding to the *Fog2 *gene deletion was absent in the *Fog2 *null sample, as was expected.

**Figure 1:**
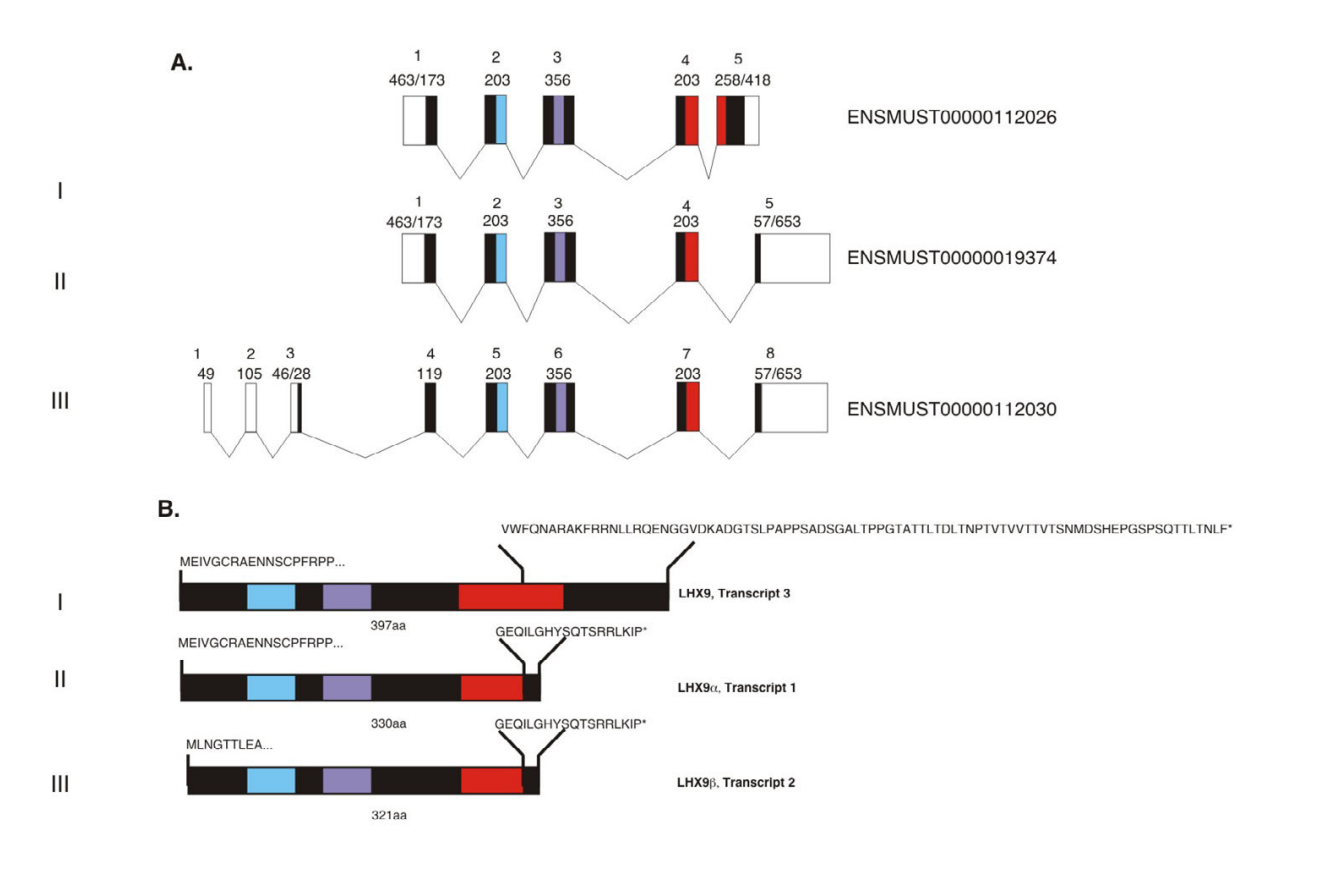
**Alternative splicing isoforms of *Lhx9***. **A**. Splicing isoforms of the mouse *Lhx9 *gene as predicted by the NCBI and *Ensembl *databases and (**B**) the proteins they encode. (I). Transcript variant 3 (NM_001042577; ENSMUST00000112026) encodes for a 397 amino acid (aa) long protein with intact homeodomain (HD); (II). Transcript variant 1 (NM_001025565; ENSMUST00000019374) encodes for a 330aa protein LHX9α with a truncated HD and a different C terminus; (III) Transcript variant 2 (NM_010714; ENSMUST00000112030) is the uncharacterized transcript and would encode for a 321aa protein LHX9β that has a different N-terminus in addition to a truncated HD. Black boxes represent coding exons; white boxes show untranslated regions; blue and purple filled boxes represent LIM domains 1 and 2; red fill shows HD; the sizes for all exons (bps) are shown for each isoform. The *Ensembl *annotation predicts much longer 3'-untranslated regions for transcripts 1 (II) and 2 (III); these, however, were not detected previously [30] and could not be confirmed by us experimentally or by a BLASTN search against the EST database (not shown).

One of the probe sets differentially represented in the control vs. *Fog2 *mutant RNA sample corresponded to the *Lhx9 *gene (~3 times; Additional File 1) encoding the LHX9 protein that belongs to the family of the LIM-HD (LIM-homeodomain) transcription factors [23]. The major research interest of this laboratory is in the transcriptional regulation of cardiac development; hence we decided to pursue the FOG2-*Lhx9 *connection. To correctly evaluate the microarray data it was critical to identify the actual mRNA species corresponding to the microarray oligonucleotide probe set. The NCBI database predicts three different mouse isoforms of *Lhx9 *mRNA; two of these isoforms have been described previously, while the third isoform has not been characterized. Originally identified transcript 3 encodes a 397 amino acid (aa) long protein with intact homeodomain (HD) that is competent to bind DNA [26,27]; this isoform is further referred to as *Lhx9-HD*. A previously described isoform, *Lhx9α*, encodes a 330 aa protein with truncated HD and a different C terminus [30]; finally, the uncharacterized transcript (variant 2) would encode a 321 aa protein that has a different N-terminus in addition to a truncated HD (Fig 1A, B). We will refer to the un-characterized isoform as *Lhx9β*.

**Figure 2:**
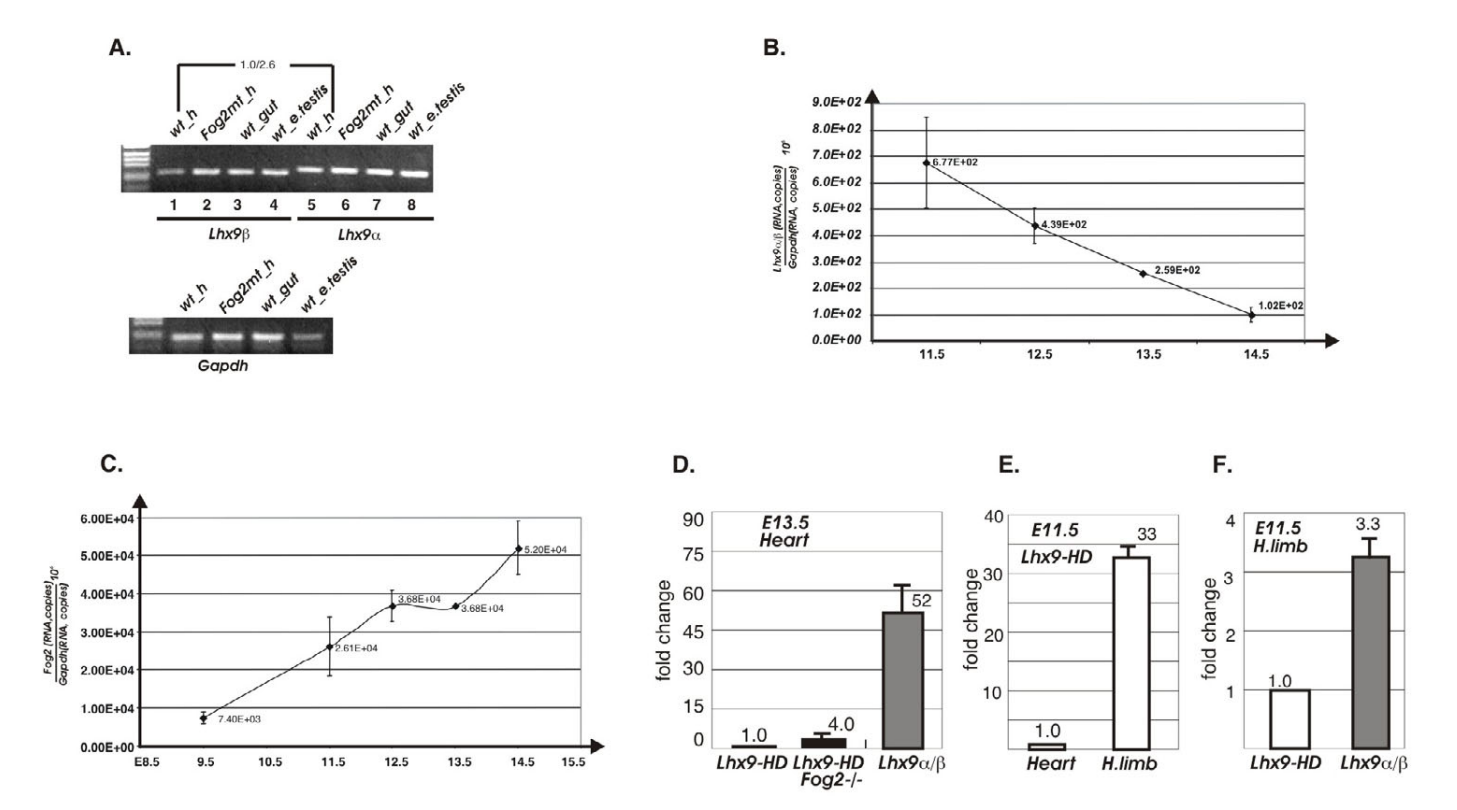
**Quantitative analysis of *Lhx9* gene expression in the control and *Fog2* null hearts**. **A**. Agarose gel electrophoresis showing RT-PCR products corresponding to the *Lhx9α *and *Lhx9β *isoforms of the *Lhx9 *gene expressed in the E11.5 tissues. The products corresponding to the cardiac tissue (lanes 1 and 5) were isolated and sequenced. Relative cardiac expression of the α(2.6) and β(1.0) *Lhx*9 isoforms in the wild-type sample was determined by the real time PCR. **(B-C)**. *Lhx9α/β *is downregulated (~7-fold) while *Fog2 *is upregulated in the wild-type E9.5–E14.5 heart as demonstrated by the real-time PCR analysis of the pan-cardiac *Lhx9α/β *(**B**) and *Fog2 *(**C**) expression. *Lhx9α/β *is not expressed in the E9.5 embryonic heart. (**D**). A comparative analysis of *Lhx9 *gene expression in the control and *Fog2 *mutant E13.5 hearts using *Lhx9-HD *and *Lhx9α/β *isoform-specific primers. The *Lhx9 *expression for both isoforms is normalized to the *Gapdh *gene expression level; the fold changes (y axis) are shown; relative expression of *Lhx9-HD *is normalized to be 1.0. (**E**). A comparative analysis of the *Lhx9-HD *isoform expression in the E11.5 hearts and hind limbs. The *Lhx9 *expression is normalized to the *Gapdh *gene expression level; the fold changes (y axis) are shown; relative expression of *Lhx9-HD *in the heart is normalized to be 1.0. (**F**). A comparative analysis of *Lhx9 *gene expression in the E11.5 hind limb using *Lhx9-HD *and *Lhx9α/β *isoform-specific primers; relative expression of *Lhx9-HD *is normalized to be 1.0.

It has been reported previously that in situ hybridization using S^35^-labeled isoform-specific RNA probes detects *Lhx9α *expression in the developing E13.5 heart, while the full-length HD-encoding *Lhx9-HD *is not detected [30]. To corroborate and extend this data we designed the primers that discriminate between all three isoforms of *Lhx9 *and performed real-time PCR analysis. By real time RT-PCR we have determined that the major *Lhx9 *isoform expressed in the developing heart at E13.5 corresponds to *Lhx9α*, while *Lhx9β *transcript is expressed at a somewhat lower level (Fig 2A). Cardiac expression of *Lhx9 *is under strict developmental control: qRT-PCR analysis of total heart RNA demonstrated that combined *Lhx9 *expression corresponding to both expressed isoforms (*Lhx9α+β*) is down-regulated between E11.5 and E14.5 approximately 7-fold (Fig 2B); during the same time period *Fog2 *gene expression is increasing (Fig 2C).

We also confirmed that a full-length isoform (*Lhx9-HD*) is scarcely detectable in the embryonic heart even when measured by a highly sensitive real time PCR assay. The cardiac expression level of *Lhx9-HD *is ~30 times (at E11.5; not shown) and ~50 times (at E13.5; Fig 2D) lower than that of *Lhx9α/β*; this data is in agreement with the previous work [30]. In contrast and as a positive control we detected *Lhx9-HD *using the same primer pair as robustly expressed in the E11.5 embryonic hind limb where its levels were ~33 times higher than in the E11.5 heart (Fig 2E); in the hind limb *Lhx9-HD *expression was only slightly lower (~3.3 times) than *Lhx9α/β *expression (Fig 2F) (see also Fig 5 for in situ hybridization analysis).

### *Lhx9* is a target of the GATA4/FOG2 transcriptional complex

**Figure 3:**
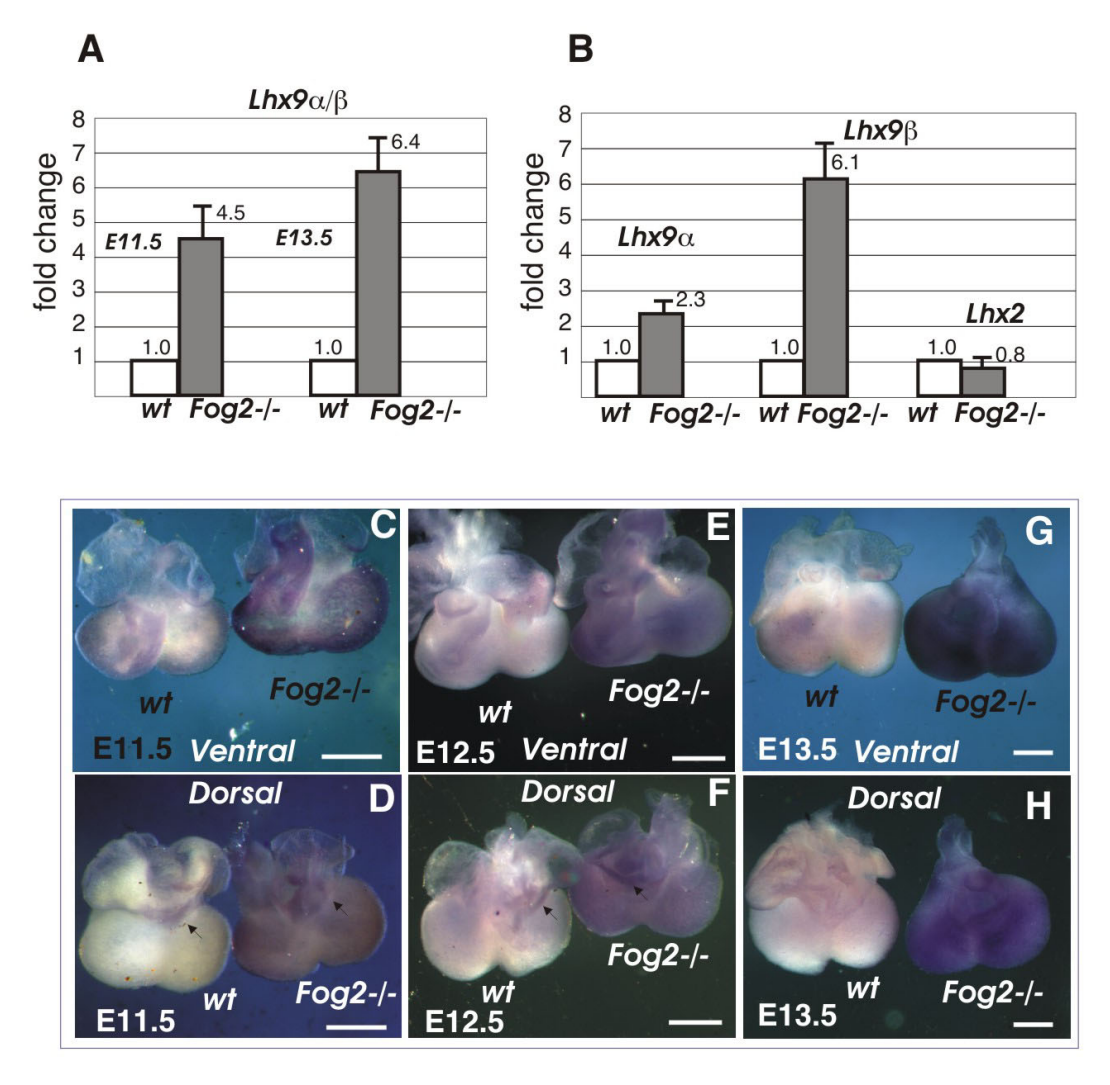
**The analysis of *Lhx9* expression in the control and mutant hearts**. **(A-B)**. *Lhx9 *gene expression is up-regulated in the *Fog2 *mutant hearts. (**A**). A comparative real-time PCR analysis of the total (*Lhx9α/β*) expression in the E11.5 and E13.5 control and *Fog2 *mutant hearts. (**B**). A comparative analysis of *Lhx9 *and *Lhx2 *gene expression in E13.5 control and *Fog2 *mutant hearts; *Lhx9 *expression is examined with α- and β isoform-specific primers. *Lhx *genes' expression is normalized to the *Gapdh *gene expression level; the fold changes (y axis) are shown; relative expression in the wild-type sample is normalized to be 1.0. (**C-H**). Whole-mount staining of the E11.5 (**C-D**), E12.5 (**E-F**) and E13.5 (**G-H**) hearts with an anti-*Lhx9α/β *RNA probe. Both ventral (**C**, **E**, **G**) and dorsal (**D**, **F**, **H**) views are shown; the wild-type sample is always on the left. In the wild-type E11.5 heart (**C-D**) ventricles, the atrioventricular groove (*arrow*) and the outflow tract are weakly positive for *Lhx9 *expression, while in the *Fog2 *null heart, *Lhx9 *expression is strongly enhanced. By E12.5 (**E-F**) and especially by E13.5 (**G-H**) *Lhx9 *expression appears almost extinguished in the control heart, while the *Fog2 *mutant sample remains strongly positive for *Lhx9 *expression. The scale bar is 200 μM.

Based on the microarray data analysis the combined value of the probe sets corresponding to *Lhx9α+β *was ~3 times higher in the E13.5 *Fog2*-null sample (Additional File 1). Real time PCR analysis confirmed the increase in *Lhx9α/β *expression in the mutant. The up-regulation is already significant at E11.5 (~4.5 excess vs. control) and becomes even more dramatic (6.4 times) at E13.5 (Fig 3A). Analysis with isoform-specific primers revealed that the overall increase in the mutant is derived mostly from the up-regulation of the *Lhx9β *isoform (6.1x), although expression of *Lhx9α *in the *Fog2 *mutant heart is also doubled (2.3x) (Fig 3B). The expression of the isoform encoding the full-length protein, *Lhx9-HD*, is also induced in the *Fog2 *mutant heart, but its level remains negligibly low (Fig 2D). In contrast, the expression of the *Lhx2 *homologue does not change in the mutant (Fig 3B).

To further compare the expression of *Lhx9 *in the control and *Fog2 *mutant cardiac samples we performed *in situ *whole-mount hybridization experiments using an anti-*Lhx9 *RNA probe that detects both isoforms (α/β). In the wild-type E11.5 heart the ventricles, the atrioventricular groove and the outflow tract are weakly positive for *Lhx9α/β *expression, while in the *Fog2 *null heart *Lhx9α/β *expression is strongly enhanced (Fig 3C–D). By E12.5 and especially by E13.5 *Lhx9α/β *expression appears almost extinguished in the control heart, while the *Fog2 *mutant sample is strongly positive for *Lhx9α/β *(Fig 3E–H). These results correlate well with the real-time PCR data (Fig 2B and Fig 3A–B). The non-cardiac expression of *Lhx9α/β *(e.g. spinal cord, limb buds and testes) remains comparable in the control and mutant embryos (Fig 5 and Additional File 2A, B; also data not shown).

### *LHX9α/β* is localized to the epicardium in the fetal heart

To establish the identity of cardiac cells that express *Lhx9α/β *we sectioned the stained hearts following the RNA in situ hybridization. As *Lhx9α/β *expression in the wild-type heart is low, we analyzed the mutant samples. We observed that *Lhx9α/β *expression is restricted to the outermost layer of the heart (not shown). To ensure that this staining is not a result of a poor probe penetration, we sectioned the wild-type and mutant E11.5 hearts and performed immunofluorescent analysis with an anti-LHX9 antibody. Among several antibodies we have tested (data not shown) only one [31] was suitable for the immunofluorescence analysis of embryonic samples. In addition to LHX9α/β this anti-LHX2/9 antibody recognizes both LHX2 and LHX9-HD; however, RNA species encoding both of these proteins are absent (i.e. *Lhx9-HD *(Fig 2D, E and [30]; *Lhx2 *[32]) from the E11.5 embryonic heart.

**Figure 4:**
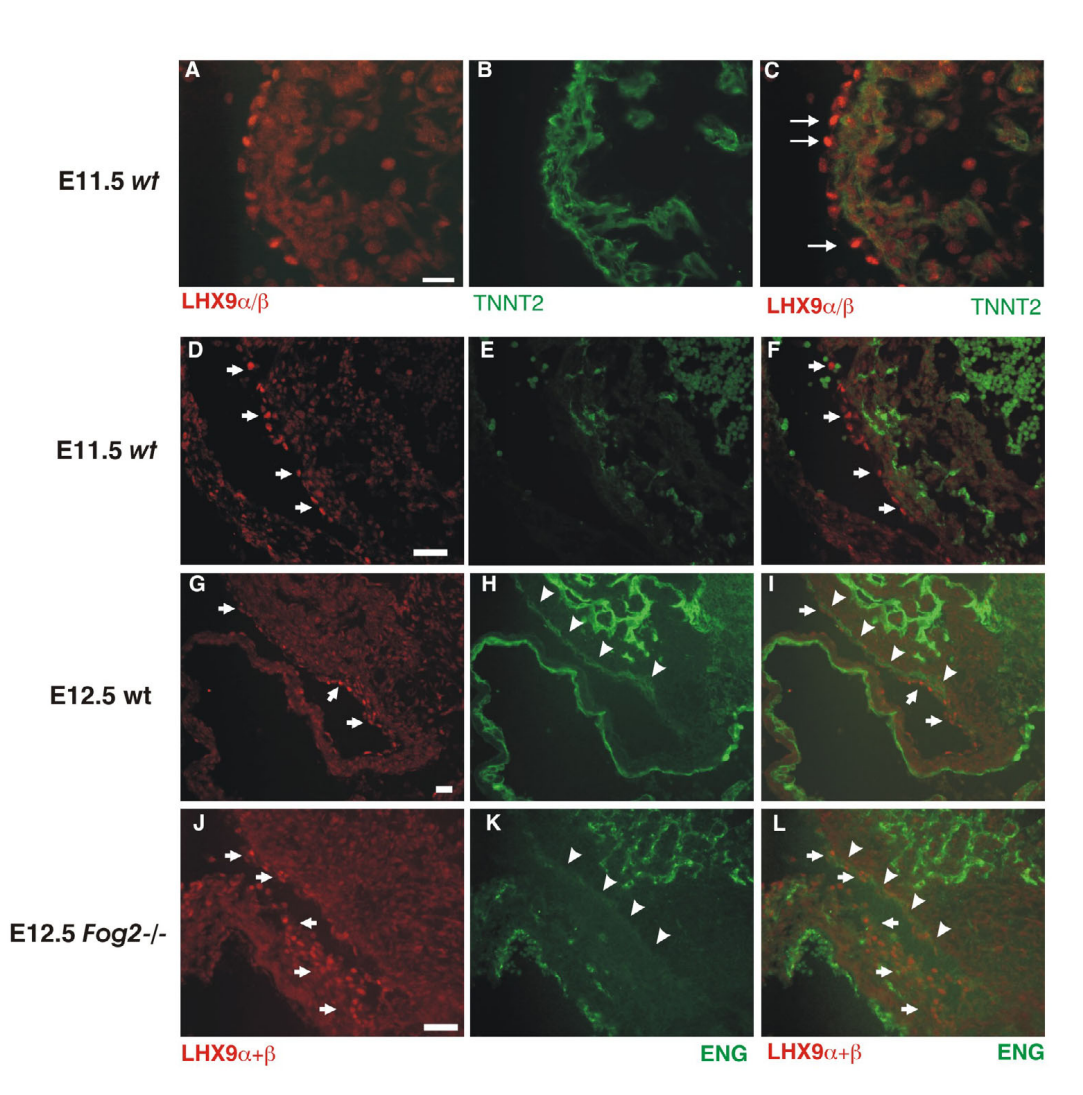
**LHX9α/β protein expression is specific to the epicardium of the heart**. **(A-C) **Immunofluorescence analysis of LHX9α/β expression in frozen sections of E11.5 heart. (A) LHX9 (red; **A **and **C**) does not co-localize with cardiac muscle marker TNNT2 (*cardiac troponin T*) (green; **B-C**). LHX9-positive cells are localized to the outmost cardiac layer (arrows). Scale bar 50 μM. **(D-L)**. LHX9α/β-positive cells (red; **D, G, J, F, I, L**) are localized to the outermost cardiac layer (*arrows*) in E11.5 (**D-F**) and E12.5 control (**G-I**) and *Fog2 *mutant (**J-L**) cardiac sections. The endothelial marker ENG (*endoglin*) (green; **E-F; H-I; K-L**) labels the epicardial cells already committed to the endothelial lineage beginning at E12.5 (arrowheads). Scale Bar: 50 μM. Control sections required longer time exposures to detect the weaker LHX9 staining in the wild-type hearts.

The antibody demonstrated strong and specific staining in all embryonic regions that were previously reported to have strong *Lhx2/Lhx9 *expression (e.g. gonads; Additional File 2C). In the heart the protein expression correlated well with the *Lhx9α+β *in situ staining pattern. LHX9α/β protein expression was detected in atria, ventricles and outflow tract; the expression was enhanced in the atrioventricular groove (Fig 4). Again, cells located in the outermost cardiac layer were the only cells that stained strongly positive for LHX9α/β. These cells (Fig 4A) stained negative for the pan-myocardial marker, TNNT2 at all times examined (Fig 4B–C) and data not shown).

To label the cells in the epicardial region at this early stage in epicardial development we used an antibody specific for the endothelial integral membrane glycoprotein *endoglin *(ENG) [33] that recognizes epicardial cells that are already committed to the endothelial lineage both in the control and in *Fog2 *mutant hearts [2]. At E11.5 the epicardial layer containing LHX9α/β-positive cells is still negative for ENG, while at E12.5 stage this endothelial marker starts to detect some (but not all) ventricular cells in the epicardial and sub-epicardial layer. We concluded that in the embryonic heart LHX9 α/β proteins are temporarily expressed in the developing epicardial cell layer. Although the majority of the LHX9-positive cells are confined to the epicardium, a weaker immunoreactivity is detectable in the myocardium (Fig 4) suggesting that a low level expression of LHX9α/β could be present in these cells.

### *Lhx9α/β* and *Lhx2*, but not *Lhx9-HD* are expressed in the proepicardium

**Figure 5:**
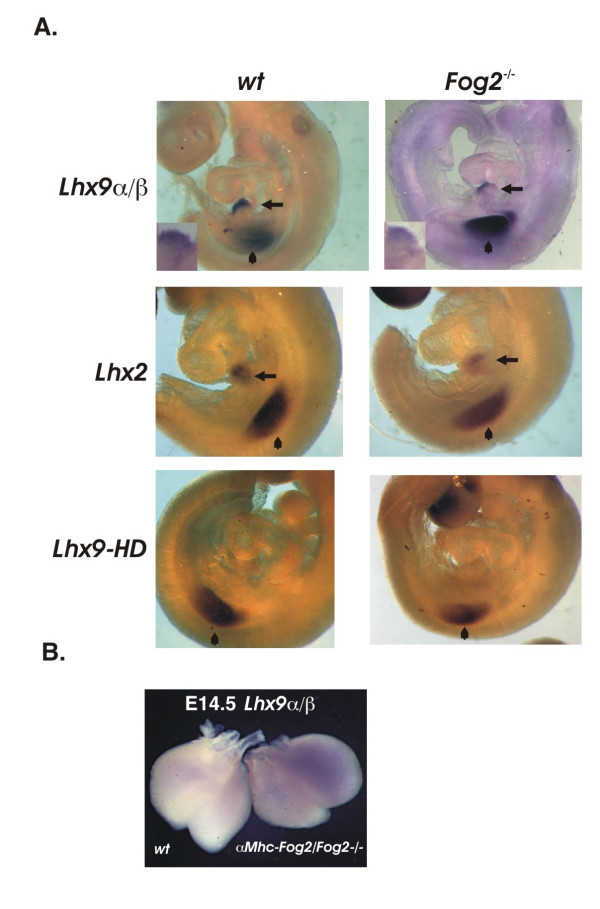
**(A) *Lhx9 α/β* and *Lhx2*, but not *Lhx9-HD* are expressed in the septum transversum in mice at E9.5**. Whole-mount in situ hybridization with anti-*Lhx9α/β *(*top panels*) and *Lhx2 *(*middle panels*) RNA probes detect a strong *Lhx9α/β *expression in septum transversum both in the control (*left panels, arrow*) and the *Fog2*^-/- ^embryo (*right panels*, arrow); an *Lhx9-HD *RNA probe does not show the expression of this isoform in the septum transversum (*bottom panels*); hindlimb bud expression is indicated by an arrowhead. The higher magnification of STM expression for *Lhx9α/β *is shown in insets (*top panels*). **(B) ***The myocardial Fog2 does not suppress Lhx9α/β expression in the heart*. Whole-mount staining of the E14.5 hearts with the anti-*Lhx9α/β *RNA probe. The wild-type sample is on the left and the *αMhc-Fog2/Fog2*^-/- ^heart is on the right. At this stage *Lhx9α/β *expression is extinguished in the wild-type heart (*left*) (also Fig. 2B) while the *Fog2 *mutant sample with the myocardial-restricted expression of *Fog2 *(*right*) remains strongly positive for *Lhx9α/β *expression.

Lineage studies in chick embryos have demonstrated that epicardium is derived from the extra-cardiac source, the proepicardium [34-38]; in mice these cells are derived (at least partially, see also [39]) from the septum transversum [40-42]. The epicardial progenitor cell cysts are formed in the proepicardial serosa at E9.5–E10.5 and cover the myocardium with a single layer of flat cells by E10.5–11.5 [43,44]. By performing an in situ hybridization for *Lhx9*α/β with E9.5 embryos we noted that *Lhx9*α/β is prominently expressed in the septum transversum in both the wild-type and *Fog2 *mutant embryos (Fig 5A, *top panels*).

Expression of the *Lhx9 *homologue, *Lhx2*, in the septum transversum (STM) has been previously reported [45]; however, we noted that the authors in their analysis used a probe that corresponds to the whole-length *Lhx2 *cDNA (NM_010710; nucleotides 460–1750). A sequence comparison revealed two large regions of almost complete identity between the *Lhx2 *probe sequence and *Lhx9*; hence, we believe the authors [45] could not discriminate between *Lhx2 *and *Lhx9 *in their in situ assay. To distinguish between these homologues we designed an *Lhx2 *probe that is limited to the most 5'-region of the *Lhx2 *sequence (NM_010710; nucleotides 42 to 488) and has no homology to *Lhx9*; we also generated an in situ probe that is specific for *Lhx9-HD*. We detected an *Lhx2 *transcript in the STM (Fig 5A, *middle panels*). In contrast, the transcript corresponding to the full-length *Lhx9-HD *could not be detected in the STM (Fig 5A, *bottom panels*); this rules out the transient role for the *Lhx9-HD *transcript in the STM. We detected no STM up-regulation of either *Lhx2 *or *Lhx9α/β *in the absence of *Fog2*; if anything, it appears that both *Lhx9 α/β *and *Lhx2 *could be expressed slightly lower in the STM of *Fog2 *mutants (e.g. Fig 5A, *insets in top panels*).

We have previously shown that transgenic expression of *Fog2 *(restricted to the myocardium with an *Mhc *promoter) extends the life of the otherwise *Fog2*-null embryos; however this rescue by myocardial-derived *Fog2 *is incomplete [2,46]. If *Lhx9α/β *expression is mostly confined to the epicardium and directly regulated there by a FOG2 protein, myocardially-driven FOG2 should not be able to repress *Lhx9α/β *expression back to its original low level. To test this we examined *Lhx9α/β *expression in the control and 'rescued' *αMhc-FOG2/Fog2*^-/- ^hearts at E14.5. Rescued hearts preserved a high level of *Lhx9α/β *compared to the control indirectly attesting to the fact that myocardial *Fog2 *is not primarily responsible for regulating *Lhx9α/β *expression (Fig 5B). This finding prompted us to examine whether FOG2 could be directly regulating *Lhx9α/β *expression as described below.

### *Lhx9* is a direct target of GATA4/FOG2 repression complex in the heart

**Figure 6:**
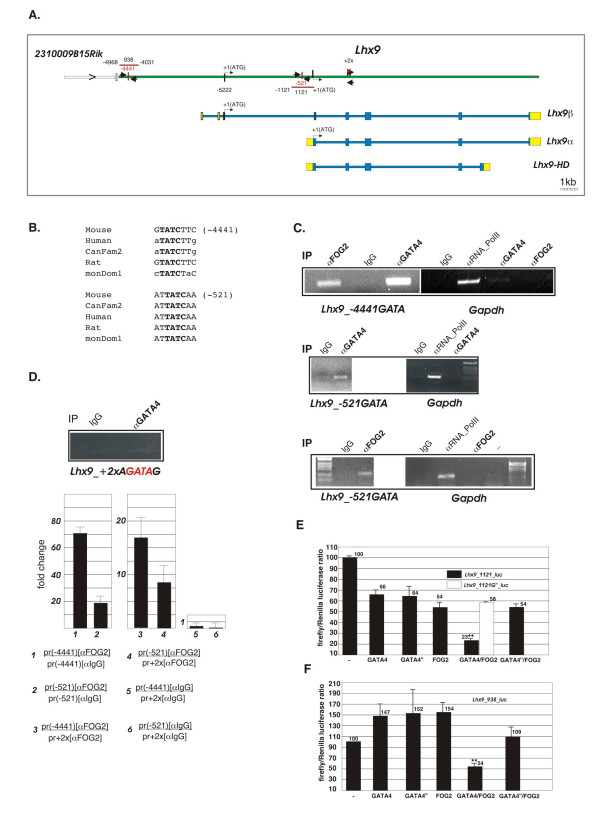
***Lhx9* gene is a direct target of the GATA4/FOG2 transcription complex**. (**A**). A map of the mouse *Lhx9 *genomic locus. Exons are shown as blue and yellow boxes; the blue boxes correspond to the protein coding regions while yellow bars depict the UTRs. Both *Lhx9 *and *2310009B15Rik *(an uncharacterized ORF flanking the *Lhx9 *locus) are oriented 5'-3' left-to-right. The *Lhx9 *introns are shown as thin blue lines. The DNA sequences corresponding to the intergenic space for all three isoforms are shown in green. The positions of the evolutionary conserved GATA sites are shown as vertical red bars and their distances with respect to the translation start sites +1(ATG) are indicated; the non-functional GATA sites are situated 3' from the start sites and ("+2x") are shown as black bars. The arrowheads are indicating the sequences amplified in the ChIP assay (see Materials and Methods for primer sequences). The horizontal red lines correspond to the DNA fragments used to generate the luciferase reporter constructs. (**B**). Alignement of the conserved reverse GATA sites (emboldened) in five genomes; the distance is shown from the first nucleotide (T) of the GATA site relative to the ATG codone of the respective isoform: β (*top*) and α (*bottom*). (**C**). Immunoprecipitations of the cross-linked chromatin from E11.5 hearts were performed with antibodies against FOG2, GATA4, RNA polymerase II or with normal IgG. Following DNA purification, samples were subjected to PCR with primers designed for the regions of the *Lhx9 *gene that contain GATA sites or the *Gapdh *promoter as a control; the PCR products were visualized on ethidium bromide gels. The PCR products were consistently observed with the total input chromatin aliquot before the immunoprecipitation step (not shown). The figure is a representative of four experiments performed.**(D) **The ChIP reactions corresponding to the α GATA4 and α IgG (same as in (**C**)) were subjected to PCR with primers designed to amplify the internal region of *Lhx9 *with two perfect consensus GATA sites (2xAGATAG;Additional File 2B) and were analyzed directly by the ethidium bromide gel (*top panel*). α FOG2 and α IgG ChIPs (same as in (**C**) were subjected to real-time PCR either with primers corresponding to the regulatory GATA-containing sequences or with internal primers. Primers corresponding to the two regulatory sequences amplify the α FOG2 ChIP reaction 70 and 18 times more efficiently than the α IgG reaction (*left panel*); they also amplify the α FOG2 reaction ~18 and 8 more efficiently than internal primers (*middle panel*); the amplification efficiency for the three sets of primers with respect to the control IgG reaction is close to 1 (*right panel*). The Y axis is showing the fold change. **(E-F) **GATA4 and FOG2 cooperate to inhibit *Lhx9 *promoters. **(E) **Luciferase reporter assays using *Lhx9*-luciferase reporters pGL3_Lhx9_1121 (black bars) or pGL3_Lhx9_1121G^m ^(white bar) or (**F**) pGL3_SV40_Lhx9_938 (black bars) in HEK 293-T cells with GATA4, FOG2 and GATA4^ki ^expression vectors. pGL3_Lhx9_1121G^m ^contains a GAAA sequence instead of the wild-type GATA sequence in position -521; the differences between the control and the mutant plasmid are not significant except for the transfection shown (the white bar). **p < 0.01, paired t-test vs control.

The loss of GATA4/FOG2 interaction leads to an over-expression (de-repression) of *Lhx9α/β *cardiac expression. Based on the inability of myocardially-restricted FOG2 to down-regulate ectopic epicardial *Lhx9α/β *expression (Fig 5B), we hypothesized that the *Lhx9 *gene could be a direct target of GATA4-FOG2 mediated repression. To demonstrate that the repression of the *Lhx9 *gene requires binding of the GATA4/FOG2 protein complex to the *Lhx9 *cis-regulatory elements we inspected the *Lhx9 *genomic locus using ECR (evolutionary conserved region) browser [47] (Additional File 3A). As both *Lhx9α *and *Lhx9β *are expressed in the heart we examined the conserved regions corresponding to both isoforms. We limited our analysis to the ~5 kb region upstream of the *Lhx9β *transcription start site; this 5 kb region is delimited by the un-characterized ORF (2310009B15Rik in the mouse; *C1ORF53 *in the human genome) (Fig 6A and Additional File 3A). We identified two evolutionary conserved GATA sites within the annotated ECRs (Fig 6A, B and Additional File 3A); one GATA site (-521) is present within the promoter region corresponding to the *Lhx9α *while the other site (-4441) is located upstream of both isoforms (Fig 6A). To examine the GATA4/FOG2 complex binding to these two elements in vivo, we performed a chromatin immunoprecipitation (ChIP) assay from E11.5 embryonic hearts. This time point was chosen since at E11.5 cardiac expression of *Lhx9 *is still present in the normal heart and is responsive to *Fog2 *expression: *Lhx9α/β *expression is strongly enhanced in the absence of *Fog2 *(Figs 2D and 3A–D). We demonstrated that either the α GATA4 or α FOG2 antibody specifically pulls down DNA containing conserved GATA sites indicating the binding of the GATA4/FOG2 complex at E11.5 (Fig 6C). As a negative control we have randomly chosen an internal region (within the *Lhx9 *gene) that contains two perfect GATA elements (AGATAG; Fig 6A and Additional File 3B). This region tested negative in the GATA and FOG2 ChIP assays (Fig 6D); we have also confirmed the specificity for primers and the FOG2 antibody by a quantitative PCR analysis of ChIP reactions (Fig 6D). We conclude that in the E11.5 heart GATA4/FOG2 complex directly binds *Lhx9 *cis-regulatory regions.

### GATA4 and FOG2 cooperate to inhibit *Lhx9* promoters

To confirm that the GATA-harboring DNA sequences upstream of the *Lhx9 *transcriptional start sites are essential for GATA4/FOG2-dependent regulation we performed luciferase reporter assays. *Lhx9 *regulatory sequences containing ECRs with ChIPed GATA sites (-4968/-4301 and -1121/+1; Fig 6A) were isolated and used to generate luciferase reporter constructs. The reporter constructs were transiently transfected into the 293 HEK cells along with the GATA4 and FOG2 expression vectors. We also generated a GATA4^ki ^expression vector (encoding for the V217G mutation in GATA4 that does not interact with FOG2 [15]). The wild-type GATA4 cooperates with FOG2 to repress *Lhx9 *promoter-driven luciferase expression while the mutant GATA4^ki ^version of GATA4 is severely impaired in this repression assay (Fig 6E, F). A luciferase construct containing a GAAA mutation in the (-521) GATA element was not co-repressed by a joint GATA4/FOG2 action (Fig 6E). We conclude that the repression of *Lhx9 *gene expression in the developing heart requires the presence of the functional GATA4/FOG2 protein complex.

### LHX9α and LHX9β interact with ISL1, but not CITED2

**Figure 7:**
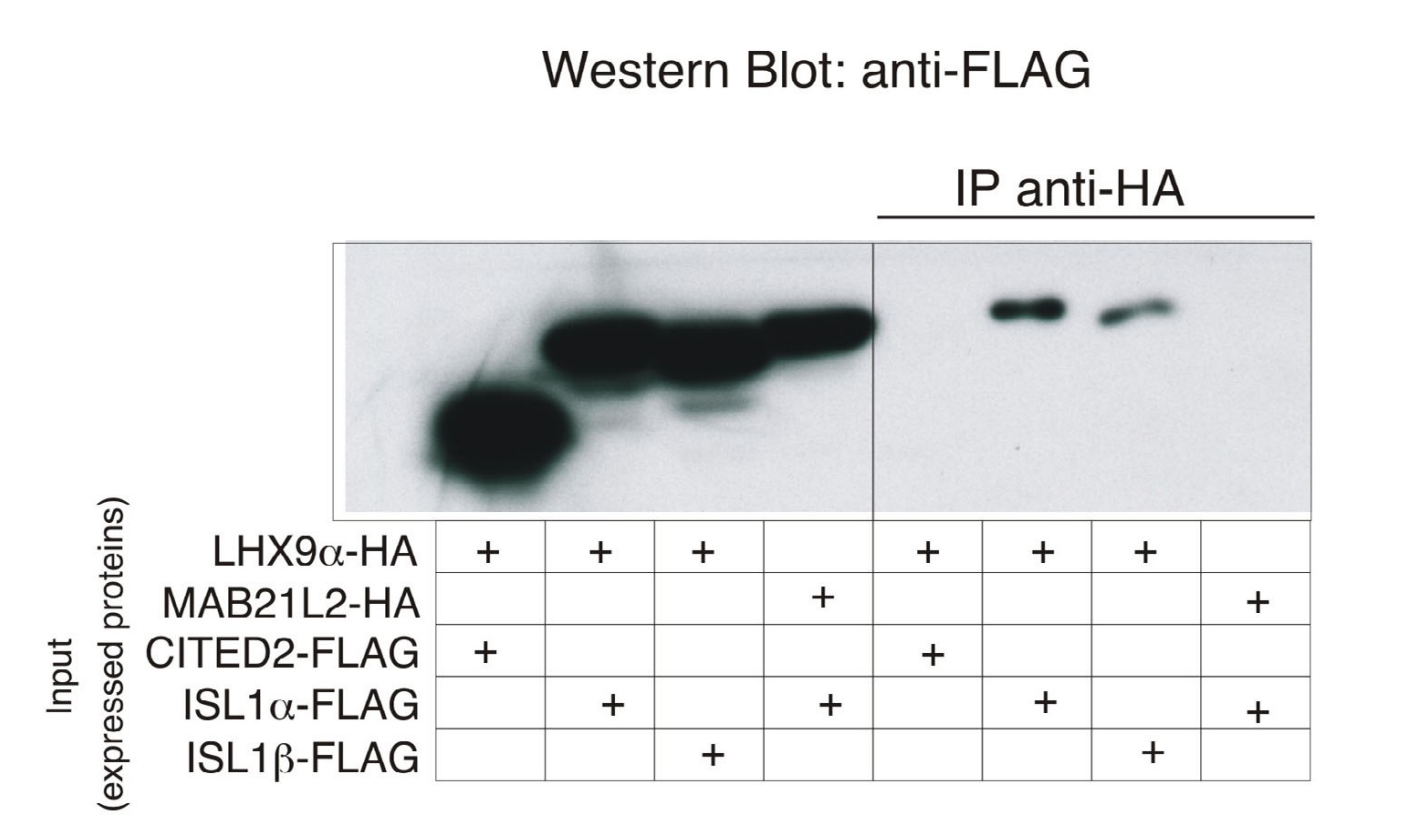
**LHX9α can interact with ISL1 isoforms, but not with CITED2**. Immunoprecipitation-Western analysis of protein interactions in the extracts of HEK293 cells transfected with DNA constructs encoding HA-tagged LHX9α and MAB21L2 (unrelated protein used as a negative control) and FLAG-tagged ISL1α, ISL1β and CITED2, as indicated.

We established that the loss of GATA4/FOG2 interaction leads to over-expression of *Lhx9α/β *in the murine heart. One of the consequences of the excessive LHX9α/β expression could be the 'trapping' of cardiac LHX factors with their interacting proteins into non-functional LHX9 α/β-containing complexes as has been previously proposed [30]. To further test this idea we probed the interactions between LHX9α and cardiac proteins that have been reported to interact with the LIM-HD factors, namely ISL1 [48,49] and CITED2/MRG1 [50]. The eukariotic expression vector encoding the epitope-tagged version of LHX9α was co-transfected with the epitope-tagged ISL1α, ISL1β or CITED2 into HEK293 cells and protein interactions were tested by immunoprecipitation-western (IP-Western) analysis. We detected the interactions between LHX9α and both iso-forms of ISl1, but not between LHX9α and CITED2 (Fig 7). This demonstrates the ability of the LHX9 isoform lacking HD to form a complex with other LIM-HD factors, such as ISL1.

### ISL1 and LHX2/LHX9 are co-expressed in the developing liver, but not epicardium or septum transversum

**Figure 8:**
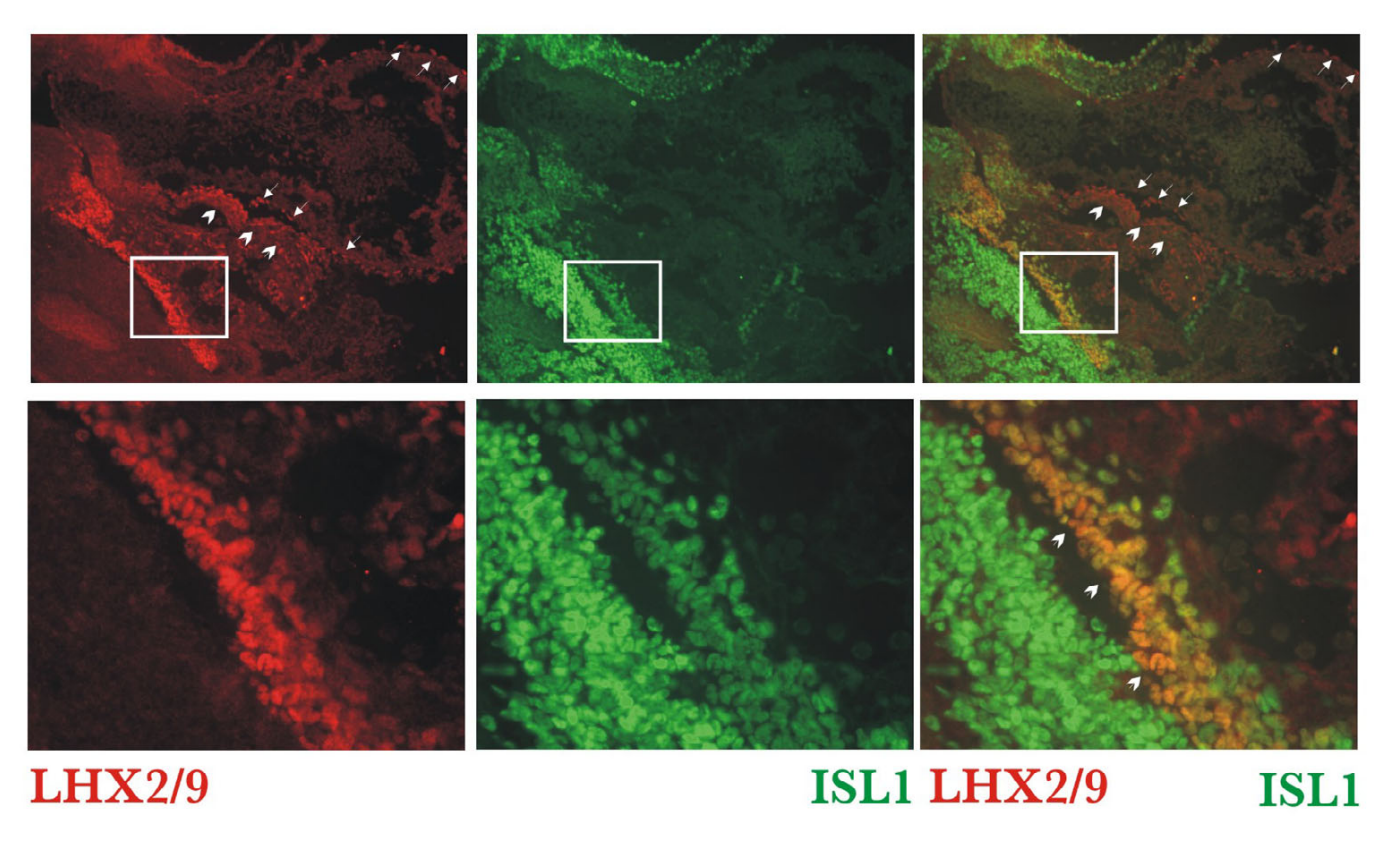
**ISL1 is co-expressed with LHX2/9 proteins in the liver primordium**. Immunofluorescence analysis of ISL1 and LHX2/9 expression in frozen sections of the E10.5 embryos. LHX2/9 staining (red) does not co-localize with ISL1 (green) in the epicardium (arrows) or septum transversum (arrowheads); the proteins are co-expressed in the liver primordium. The bottom panels are higher magnifications of the areas within white rectangles (top).

Previous lineage studies in chick embryos have demonstrated that the smooth muscle of the coronary vessels derives from the proepicardium; similarly, in the mouse these cells are derived from the septum transversum. LHX9α/β is prominently expressed in the STM at E9.5 and in the epicardium starting at E11.5 (Figs 3 and 5). Recent lineage tracing analysis in Isl1-Cre mice has demonstrated that the progeny of Isl1-expressing cells contributes to endothelial and vascular smooth muscle lineages, specifically the smooth muscle of coronary vasculature [51]. As our IP-Western analysis showed that LHX9α can interact with ISL1 (Fig 7) we sought to determine whether LHX2/LHX9α/β and ISL1 were co-expressed in the epicardium or septum transversum and performed double-fluorescence labeling experiments in E10.5 embryos. We were unable to co-localize ISL1 to LHX9 α/β-positive epicardial cells (Fig 8, top panels; arrows); this data is in agreement with the reported absence of ISL1 expression in the epicardium [51]. Similarly, cells in the septum transversum that stained positive with an anti-LHX2/LHX9 antibody were also ISL1-negative (Fig 8, top panels; arrowheads); we, however, detected a subset of cells that co-express ISL1 and LHX2/9 in the developing liver (Fig 8, bottom panels; arrowheads). Expression of *Lhx9 *in the developing liver has been previously reported [27].

## Discussion

Recent evidence suggests that different splicing isoforms of the same transcription factor may have competing/opposing as well as unrelated roles in cellular differentiation (e.g., [52-54]). We have demonstrated that GATA4/FOG2 transcription complex is essential for the repression of *Lhx9 *gene transcription in cardiac development; we have also determined that the *Lhx9 *isoform encoding for the protein with intact homeodomain is not present in the embryonic heart, while both α and β isoforms encoding a truncated homeodomain are expressed. This differential expression of *Lhx9 *isoforms suggests that truncated LHX9 proteins have a separate function independent of the full-length LHX9 molecule. This assertion is supported by the previously reported observation that LHX9 isoforms do not directly compete with each other and instead function in different pathways during neuronal differentiation [55].

One of the objectives of this study was to identify the downstream targets of GATA4/FOG2 regulation in various sub-compartments of the developing heart and to understand how the mis-regulation of these targets contributes to severe cardiac defects in *Fog2*-null and *Gata4*^*ki/ki *^embryos. We have now determined that *Lhx9α/β *is a target of GATA4/FOG2 repression, with wild-type hearts down-regulating *Lhx9α/β *epicardial expression starting at least on E11.5, while hearts deficient in FOG2 fail to do so (Fig 3). The inability of the *Fog2*-null heart to down-regulate *Lhx9α/β *expression may be a contributing factor in the constellation of the cardiac abnormalities caused by *Fog2 *deficiency [2]. Although this study mostly focused on the *Fog2 *mutants, real-time PCR analysis with the E12.5 *Gata4*^*ki/ki *^hearts [15] also revealed a significant, albeit weaker, up-regulation of the *Lhx9α/β *gene expression in this mutant (~3 times; data not shown). This more modest up-regulation in the *Gata4*^*ki *^mutant could be due to partial compensation by the other *Gata *family member, *Gata6*, that is expressed in the developing heart.

We also demonstrated here that *Lhx9α/β *expression is initiated in the septum transversum (Fig 5) and that these are the epicardial cells that continue expressing LHX9*α/β *in E11.5 hearts and downregulate this expression shortly thereafter. Further experiments will confirm whether LHX9-positive epicardial cells are direct descendants of the septum transversum cells. The requirement to down-regulate *Lhx9 *expression is no longer satisfied in the epicardium of the *Fog2*-null mutant hearts (Fig 3). We propose that abnormally high level of *Lhx9 *gene expression that is characteristic of an earlier stage in epicardial cell development is likely to be a contributing factor in the impaired differentiation of the epicardially derived cells in the *Fog2 *mutant embryos [2].

Chromatin immunoprecipitation (ChIP) confirmed the roles of the two evolutionary conserved GATA sites in *Lhx9 *gene cis-regulatory elements. Since DNA complexes were precipitating with either anti-GATA4 or anti-FOG2 antibodies, this assay demonstrated that in the E11.5 heart these sites are occupied by the GATA4/FOG2 complex rather than GATA4 alone. We could no longer detect GATA4/FOG2 complex binding to these sites in the E14.5 hearts (not shown) suggesting that *Lhx9 *repression at this later stage of cardiac development is likely to be GATA4/FOG2-independent. In agreement with the ChiP data, luciferase reporter assays confirmed the cooperative repression by GATA4 and FOG2 (Fig 6E, F). Although GATA sites have been previously identified in the proximal promoters of *Anf*, *Bnp *and α-*Mhc *genes, these genes' expression is not affected by the GATA4/FOG2 interaction loss. It is possible that other transcription factors regulating *Lhx9 *expression ensure the selectivity of GATA4/FOG2 binding; however, computer analysis of ECRs harboring GATA sites did not reveal sequence conservation for any other transcription factors in these regions. *Lhx9 *gene expression is restricted to a limited number of tissues during development and is likely to be tightly regulated (e.g. [26,27,31]). Transcriptional regulation of *Lhx9 *is not well understood and the crosstalk between the (yet unknown) activators of *Lhx9 *expression and its repressors, GATA4/FOG2, remains to be elucidated. In this respect we also cannot exclude the contribution of an indirect regulation mechanism where GATA4/FOG2 would normally activate a yet unknown repressor X of *Lhx9 *transcription; *Fog2 *loss in this case will result in down-regulation of X and de-repression of *Lhx9*. In summary, although our ChIPs as well as transient transfection data argues in favor of a direct repression mechanism, it is possible that, once other trans-acting factors governing *Lhx9 *regulation are uncovered, they will be also deregulated in the *Fog2 *knockout.

We have previously shown that transgenic expression of *Fog2 *restricted to the myocardium with *αMhc *promoter extends the life of the otherwise *Fog2*-null embryos; however this rescue by myocardial-derived *Fog2 *is incomplete [2,46]. In this respect, increased level of *Lhx9 *expression still persists in the E14.5 hearts of the 'rescued' *αMhc-Fog2/Fog2*^-/-^mutants (Fig 5B) indirectly attesting to the fact that myocardial *Fog2 *is not responsible for regulating *Lhx9α/β *expression. It is likely that the inability to down-regulate *Lhx9 *in the epicardium is, at least partially, responsible for the incomplete rescue in the *αMhc-Fog2/Fog2*^-/- ^mutants. Importantly, in a sensitive qRT-PCR the level of *Lhx9 *expression in *αMhc-Fog2/Fog2*^-/- ^hearts was slightly reduced (not shown) suggesting that a low level myocardial *Lhx9/LHX9 *expression is present and controlled by GATA4/FOG2.

Loss of GATA4/FOG2 interaction has a profound early effect on testis differentiation [46]; similarly, the *Lhx9 *gene is expressed in the developing gonads and *Lhx9 *gene targeting that removes two exons encoding for the LIM domains results in an early gonadal defect [29]. However, in contrast to the situation in cardiac development, we do not observe an up-regulation of *Lhx9 *gene expression in the gonads of GATA4/FOG2 mutants (e.g., Additional File 2) suggesting that GATA4 and FOG2 do not control *Lhx9 *in this tissue.

Currently, the function of LIM-HD factors in cardiac development (with the notable exception of ISL1) is not well understood. Computer analysis of gene expression database (as well as our own microarray analysis, data not shown) shows that *Lhx6 *is the only other member of the *Lhx *family that is expressed in the developing heart in the amount comparable to *Lhx9α/β*; no cardiac defect for *Lhx6 *loss of function has been reported [56].

Our data confirms and extends the previous observation that *Lhx9 *splicing isoforms are expressed in a tissue-specific manner [30]. We also demonstrated that these truncated *Lhx9 *transcripts lead to the expression of the protein; this is an important confirmation since an appearance of an RNA encoding for a regulatory factor does not always correlate with protein accumulation (e.g. *Myf5 *RNA and not the protein are expressed in neurons [57]). It was proposed that *Lhx9α *encodes for a protein with a truncated HD that can compete with LHX9 (or other LIM-HD factors) for limited amounts of nuclear CLIM cofactors like Ldb1 [55]; *Ldb1 *knockout animals do not develop heart anlage [58]. CLIM cofactors can dimerize and interact with two LIM-HD proteins at the same time ([48,59]; reviewed in [60]). In addition to the simple sequestration of Ldb1, the adjustment of stoichiometry between various CLIM-LHX complexes can involve differential degradation of some, but not other complexes [61,62]. Finally, one of the consequences of the excessive LHX9α/β-Ldb1 complex formation could be 'trapping' of cardiac LHX factors with their interacting proteins into non-functional LHX9 α/β-containing complexes. Our IP-Western analysis demonstrated that both LHX9α and LHXβ could interact with other LIM-HD proteins such as ISL1 (Fig 7), suggesting that LHX9 α/β could function in mammalian cells as part of the multi-protein complexes. The ISL1 protein, however, is unlikely to serve as a partner for LHX9α/β in the epicardium or septum transversum as cells expressing LHX2/9 in these tissues do not express ISL1 (Fig 8).

In summary, our data suggest that the function of the LHX9α/β protein during the proepicardial development should be evaluated. This function maybe masked by other *Lhx *family members or by unrelated compensatory mechanisms and hence not revealed in the *Lhx9*-null animals [29]. We also provide evidence that GATA4/FOG2 regulate *Lhx9 *gene expression directly through binding to the evolutionary conserved GATA sites in the *Lhx9 *regulatory regions. The loss of GATA4/FOG2 interaction leads to de-repression of the *LHX9α/β *expression in epicardial cells; this abnormally high expression may account for some of the cardiac malformations observed in the *Gata4*^*ki/ki *^and *Fog2 *null mutants.

## Conclusion

LHX9 belongs to the family of the LIM-HD (LIM-homeodomain) transcription factors. We have now determined that the developing mouse heart normally expresses truncated isoforms of *Lhx9 *– *Lhx9α *and *Lhx9β*. Whereas, the expression of the *Lhx9 *isoform that encodes a protein with an intact homeodomain is extremely low. At E9.5 *Lhx9α/β *expression is prominent in the epicardial primordium, septum transversum; in the E11.5 heart LHX9-positive cells are localized to the epicardial mesothelium. Thereafter in the control hearts *Lhx9α/β *epicardial expression is promptly down-regulated; in contrast, mouse mutants with *Fog2 *gene loss fail to repress *Lhx9α/β *expression. Chromatin immunoprecipitation from the E11.5 hearts established the roles of the two evolutionary conserved GATA sites in *Lhx9 *gene cis-regulatory elements. In transient transfection studies the expression driven by the cis-regulatory regions of *Lhx9 *was repressed by FOG2 in the presence of intact GATA4, but not the GATA4^*ki *^mutant that is impaired in its ability to bind FOG2. This study identifies the first direct target for the GATA4/FOG2 repressor complex in the heart, *Lhx9α/β*.

## Methods

### Mouse strains and genotyping

Previously described *Fog2*^+/- ^[2] and *Gata*^*ki*/+ ^[15] animals were bred onto a pure C57Bl/6 background (a kind gift of Dr. Eva Eicher, Jackson laboratory); *αMhc-Fog2 *animals have been previously described [2,46]. Mice were genotyped by PCR using genomic DNA prepared from tail snips. *Fog2*^- ^null and wild-type alleles were distinguished by PCR using the following primers:

F2gen12: GCTCCAGACTGCCTTGGGAAAAG; F2gen13: CCACAGCAAGAGAACATTTCCCAGAATACC, F2gen14 GCTGCATGTGATGAGCAATAAAACTTCTTG; GATA4 knock-in allele was detected as described previously [63] by using primers GkiF TGCGGAAGGAGGGGATTCAAAC and GkiR TCTGAGAGAACTGAGGGGGTTAGC. The presence of the *αMhc-Fog2 *transgene was determined by using primers FP7 AGCGAGCGGAACCTGCAAG and FP9 TGTAGTTACAGACCGTGCA. All animal protocols have been approved by the Dartmouth Animal Care and Use Committee.

### Microarray analysis

The total RNA from control and mutant (*Fog2 *null) E13.5 hearts was applied to Affymetrix microarray (Dartmouth Genomic and Microarray laboratory); microarray data was analyzed using the Gene Traffic (Iobion Informatics) program.

### Plasmids

**pCS2+Gata4 **and **pCS2+Fog2 **were previously described [1]. To generate the pCS2+ Gata4^ki ^mutant vector we introduced the V217G mutation [15] by PCR using the following primers: GCAGAGAGTGTGGCAATTGTGG (forward) and CCACAATTGCCACACTCTCTGC (reverse).

**pCS2+HA_Lhx9α**. The full-length cDNA (993 bps) encoding for the LHX9α isoform was generated from the total cDNA prepared from E11.5 embryonic hearts using the First Strand cDNA Synthesis Kit (Invitrogen). The PCR fragment corresponding to the *Lhx9a *cDNA was generated with the following primers: Lhx9α-FL_BamHI-F GGATCCCAATGGAAATAGTGGGGTGCCGAGCC and reverse Lhx9α-FL-XhoI-R 5'-CTCGAGTAAGGGAATTTTCAAACGTCGGGAT. An insert was isolated by the BamHI/XhoI digest and cloned into the pCS2+ vector containing a HA tag (unpublished).

**pCMV5a-Isl1α_FLAG **and **pCMV5a-Isl1β_FLAG **plasmids were generated essentially as described above for pCS2+HA_Lhx9α. PCR fragments corresponding to the full-length Isl1α (1050 bp) or Isl1β (981 bp) cDNAs were generated using the following primers Isl1_FL_BamHI_F 5'-GGATCCATGGGAGACATGGGCGATCC and reverse Isl1_FL_BglII_R 5'-AGATCTTGATGCCTCAATAGGACTGGCTA; inserts corresponding to both isoforms were excised with BamHI/BglII and cloned into a pCMV5a vector containing the C-terminal FLAG tag (Sigma).

**pCMV5a-Cited2_FLAG **was generated using a commercially available plasmid (IMAGE clone 6415181; Open Biosystems) as a template. A PCR fragment corresponding to the full length *Cited2 *cDNA (810 bps) was obtained with the following primers: Cited2-fl-BamHIF 5'-GGATCCATGGCAGACCATATGATGGCCATGAA and reverse Cited2-fl-BglII-R 5'-AGATCTTGAACAGCTGACTCTGCTGGGCTGC; an insert was excised with BamHI/BglII and cloned into pCMV5a.

**pGL3_lhx9_1121 and pGL3_lhx9_1121**^Gm^: The promoter region (bps -1121 to -1 from the ATG codon of the *Lhx9α *isoform) was isolated by PCR using genomic DNA from CJ7 ES cells as a template. The following primers were used: Lhx9_1121_KpnI-F GGTACCTTCACTTTGGTGGACGTCTCAGAGC (forward; a KpnI site is underlined) and Lhx9_1121_NcoI-R CCATGGAAACACACGCCTGGGGCTCTCAGTT (reverse; a NcoI site is underlined). A PCR-generated fragment was cloned into the pSC-A vector (Stratagene); KpnI/NcoI fragment containing the *Lhx9 *gene fragment was isolated and inserted into pGL3_Basic vector (Promega) containing the luciferase reporter gene. The mutant version of this promoter, 1121^Gm^, disrupted a GATA site positioned at -521 upstream from the ATG codone. The following primers were used to insert a mutation: CTCCTACTAAATTTTCAAAAATGG (forward) and CCATTTTTGAAAATTTAGTAGGAG (reverse); the TATC/GATA site was replaced with the TTTC/GAAA sequence (the altered sequence is underlined).

**pGL3_SV40_lhx9_938: **The enhancer region from -4968 to -4031 bp upstream of the ATG codon of the *Lhx9β *isoform was isolated by PCR essentially as described above. The following primers were used: Lhx9_938_KpnI-F GGTACCTCCATATGGCCAAGTCAATGTGA (forward; a KpnI site is underlined) and Lhx9_938_NcoI-R CCATGGTGAGCAGCAGCTTCCTGTTACTG (reverse; a NcoI site is underlined). A PCR-generated fragment was introduced into the pGL3_SV40 vector (Promega) containing the luciferase reporter gene and the minimal SV40 promoter.

The PCR fragments incorporated into DNA constructs were verified by sequencing.

### Total RNA extraction

Hearts (both ventricles and atria) were dissected from the embryos and preserved in RNAlater (Ambion) until further use. Hearts were disrupted by forcing the tissue through a 26 gauge needle. Total RNA was prepared using RNAeasy columns (Qiagen) according to the manufacturer's protocol. On average 1.5–2 μg of total RNA was obtained from one E13.5 heart. RNA concentration was determined by measuring absorbance at 260 nm; RNA was stored at -80°C. Hind limb RNA was prepared following essentially the same protocol, except that no shredding of the tissue was necessary.

### Quantitative Real Time RT-PCR

cDNA was prepared from 1–2 μgs of total cardiac RNA with a First Strand cDNA synthesis kit (Invitrogen). Real-time PCR was performed using a PCR SYBR Green I Kit (Applied Biosystems) according to the manufacturer's instructions in a 7500 Fast Real-Time PCR system machine and a standard amplification protocol was established for each gene. Calibration curves were generated essentially as described before [64]. Values from at least three independent experiments were compared; standard deviation was calculated using Excel (Microsoft) application.

The following primers were used for detection of *Fog2 *and *Lhx9 *isoforms (See table 1).

**Table 1:** 

Primer	forward 5'♦ 3'	reverse 5'♦ 3'	Accession number	size
*Fog2*_	**CGCCTTTGTGGTGGACTTTGACT**	**GCTTCTCGTTGCCTCCCACTACA**	NM_011766	250
*Gapdh*	GCTCACTGGCATGGCCTTCCGTG	CGGAAGAGTGGGAAGAGACA	NM_001001303	200
*Lhx9-HD*	ACAAATTTGGTCTGCAAGATGTATT	AGGAATATAATTCGCCCATCGTAAT	NM_001042577	171
*Lhx9α/β*	CAGAAGACCAAACGGATGCGAAC	TAGGGAATTTTCAAACGTCGGGATGTT	NM_001025565	198
Lhx9α	ATGGAAATAGTGGGGTGCCGAGCC	GTCTACGGCCAGCAGATAGTACCT	NM_001025565	264
Lhx9β	ATGCTGAACGGCACCACTCTAGAGG	GTCTACGGCCAGCAGATAGTACCT	NM_010714.	237
*Lhx2*	ACCACCAGCTTCGGACAATGA	GCCCGTGTTTTCCTGCCGTAA	NM_010710	172

### Whole-mount in situ hybridization

The 504 bps *Lhx9α/β *probe corresponds to the 3'-untranslated region of mouse *Lhx9 *RNA, +1146 to +1650 from the ATG codone [30]. The probe region was amplified by PCR using total cardiac E11.5 cDNA as a template with the following primers: lhx9-1146-F GAGTGAGACATAAGTGTCATT (forward) and the lhx9-1650-R AATGTTTGCATCAAATAAAATG (reverse). The 517 bps *Lhx9-HD *probe corresponds to the + 1054 to +1571 from the ATG codone of the isoform encoding for the HD-containing protein (NM_001042577; [30] of *Lhx9*; the region was amplified using the primers: lhx9-1054-F: CTCTCACTCCACCCGGCACTG and lhx9-1571-R: AGGAATATAATTCGCCCATCGTAAT. Finally, the 466 bps *Lhx2 *probe corresponded to the 5'-region (-425 to +21 from the ATG codone) of *Lhx2 *(NM_010710); the primers used were lhx2-425F: CACCTAGCTGTTCCTGGGTGAAC and lhx2-21R: CCGACAGACTGTGGAACAGCATC. The PCR fragments were cloned into the pSC-A vector (Stratagene) and the digoxigenin-labelled RNA probes were generated according to standard procedures [65]. *In situ *hybridization and staining were performed as previously described [2]. For each embryonic (E) time point at least two independent experiments were done. Stained hearts or embryos were re-fixed in 4% paraformaldehyde, washed and kept in PBS. Images were acquired, processed and mounted as previously described [66].

### Chromatin Immunoprecipitation (ChIP)

For ChIP experiment hearts were dissected out in ice-cold PBS; eighteen E11.5 or three E14.5 mouse hearts were pooled. Tissue was chopped with a sharp razor blade and fixed in a freshly prepared 1% paraformaldehyde. Chromatin extraction and immunoprecipitations were performed using an EZ ChIP assay kit (Upstate) according to manufacturer's protocol. Chromatin was shared 6 × 10 sec using the sonicator (MODEL 250, Branson) at 40% duty cycle. Shared chromatin was pulled down either with the goat anti-mouse GATA4 or goat anti-mouse FOG2 antibody (both from Santa Cruz); the control anti-mouse RNA polymerase II, clone CTD4H8 antibody (Upstate); or the control mouse IgG (Upstate). PCR products were recovered by PCR Purification Kit (QIAGEN) PCRs were done with Hotstart Taq DNA polymerase set (Qiagen) using initial denaturation 95°C for 10 min (to activate Tag polymerase) followed by 30 cycles of denaturation (94°C; 20 sec), annealing (55°C; 30 sec) and extension (72°C; 30 sec) with the final extension at 72°C for 5 min. The following PCR primers were used to amplify the regions of *Lhx9 *or *Gapdh *genes (See table 2).

**Table 2:** 

*Gene Regions*	Sequence F5'♦ 3'F	Sequence F5'♦ 3'R	size
*Lhx9*_-521ChIP	GAGATTTCCTTAGCTTTGCGTC	ACACAAAGAAAGAGAACGAAGTGC	229
*Lhx9*_-4441ChIP	GAGATTTCCTTAGCTTTGCGTC	CTATAGAGGGAGAATGAAGAACGGTC	265
*Gapdh*_ChIP	GTCACCTCCTGAGCGGGGCAATCTC	CCCGCCTCCCGCCCTGCTTATCCAG	212
*Lhx9*+2X_CHIP	AGGTACTATCTGCTGGCCGTAGAC	CAAGGGACAGAGAAGGGACTCCGGATT	246

PCR products were recovered from agarose gels and their nucleotide sequences were confirmed.

### Cell Culture and Transient Transfection Assays

#### Cells

293T HEK cells were maintained in DMEM media (Mediatech) with 10% newborn calf serum (NCS) and antibiotics (DMEM/NCS).

#### Transfection

Transient transfections were performed using a standard HBS × CaCl_2 _protocol [67]. 0.1–0.3 μg of Luciferase reporter plasmids and 0.1–0.3 μg Renila plasmids were combined with 2–3 μg of GATA4/GATA4^ki ^or FOG2 expressing plasmids or 2–3 μg of a balancer plasmid. Cells from one confluent well of a 6-well plate were used; cells were incubated with the precipitate overnight and the media was replaced with a fresh aliquot of DMEM/NCS the next morning.

#### Luciferase Dual Reporter assay

Cells were lysed 48 hours post-transfection as recommended by the Luciferase Dual Reporter Assay protocol (Promega). After lysis the cells were centrifugated at maximal speed, the supernatant was diluted 10 times and used immediately for measuring the luciferase activity; activity of the firefly luciferase was normalised to the Renilla reporter activity. Each experiment was repeated at least three times. Each value was compared to control (reporter vectors in the presence of the balancer plasmid) in each experiment and values from three independent experiments were analyzed; the final graph was built using Microsoft Excel application (Microsoft).

### Immunofluorescence Analysis

Embryos were fixed in 4% paraformaldehyde in PBS for 2 h (hours), washed with PBS for 4 h in a cold room using several changes of PBS. Fixed embryos were soaked in 30% sucrose in PBS overnight at 4°C, positioned and frozen in the OCT (Fisher Scientific) and kept at -80°C until further use. 10 μM sections of the frozen embryos were cut on the cryostat (Leica) and lifted on the Superfrost slides pre-treated with the Vectabond (Vector). For the immunodetection of the LHX9 protein, slides were blocked/permeabilized in PBS containing 5% Carnation non-fat milk and 0.1% Triton X-100 (Fisher) for 1 h at RT. Blocked slides were reacted with the rabbit anti-LH2A/B antibody (a kind gift from Dr. Thomas Jessell) diluted 1:300 and detected with donkey anti-rabbit Alexa 555 antibodies (Molecular Probes). Mouse anti-Isl1 39.4D5b diluted 1:300 was detected by goat anti mouse Alexa 488 (Molecular Probes). Mouse anti-TNNT2 (1:500; USBiologicals) was detected with goat anti-mouse Alexa 488; rat anti-Endoglin (1:300; Pharmingen) was detected with the goat anti-rat Alexa 488 antibody; all secondary antibodies were used at a 1:500 dilution. PBS-washed slides were mounted in Vectashield media with DAPI (Vector). Pictures were acquired using a Magnafire camera (Olympus) with an Olympus fluorescent microscope as previously described [66].

#### Immunoprecipitation (IP)

Transient transfections were performed using an HBS × CaCl_2 _protocol essentially as described above with HEK293 cells cultured on 10 cm plates with 9 μg of pCS2+HA-Lhx9 combined with 9 μg pCMV5a-Isl1α_FLAG or pCMV5a-Isl1β_FLAG or pCMV5a-Cited2_FLAG or pCMV5a-Mab21l2_FLAG plasmid (negative control). Cells were harvested 48 hours after transfection as previously described [1]. Briefly, plates were washed with PBS put on ice and cells were lysed directly with a "TNN-plus" buffer [50 M Tris HCl pH7.5, 150 mM NaCl, 0.5% Igepal (Sigma), 5 mM EDTA and a complete protease inhibitor (Roche)]. Lysates were centrifuged and supernatants were transferred to new tubes several times to remove cell debris. IP was performed by incubating the cleared lysates with 25 μl of Anti-HA Affinity Matrix (Roche) for 2 h in a cold room followed by 4 washes with TNN-plus. 1/100 of a lysate or 1/10 of an IP were analysed by a Western blot. Proteins were detected on a Immobilon membrane (Millipore) either with a rabbit HA antibody (Santa Cruz) followed by an anti-rabbit HRP conjugate (Bio-Rad; 1:3000) or directly with an anti FlagM2 peroxidase Conjugate (Sigma; 1:3000).

## Authors' contributions

FOS designed and carried out most of the experiments and wrote the initial draft of the 'methods' section of the manuscript (*ms*). NLM generated and genotyped embryonic samples, performed some real-time PCRs and participated in the interpretation of the results and in the writing of the *ms*. LLL carried out several qRT-PCRs, some transient transfection experiments and in situ hybridizations. SGT and FOS together conceived and coordinated the study, interpreted the data and edited the *ms *and the referee correspondence. SGT participated in the mutant analysis, assembled figure panels and wrote, edited and revised the *ms *and the referee correspondence. All authors read and approved the final *ms*.

## Supplementary Material

Additional file 1 Differentially Expressed Genes in E13.5 Control vs. Mutant Hearts Click here for file

Additional file 2 (A-B) An E13.5 XY gonad from the control (A) and *Fog2*^-/- ^mutant (B) embryos were examined by in situ hybridization with an *Lhx9α/β *RNA probe. The mutant sample lacks the sex cords resulting from a block in male sexual development [46]; g-gonad, m-mesonephros. (C) A specific staining in the genital ridge of the E11.5 *Fog2*^-/- ^embryo with LHX2/9 antibody [31]. Click here for file

Additional file 3 (**A**) *In silico *identification of the putative GATA elements in the *Lhx9 *gene locus. An ECR browser output showing the conservation profiles of the human region in comparison with the monkey, mouse, rat, dog and opossum genomes. Exons are shown in blue and yellow; the blue bars correspond to the protein coding regions while yellow bars depict the UTRs. The 5'-3' orientation of the gene is shown by the blue arrow lines on top. Dark red bars show the distribution of ECRs (the areas of the respective genome with greater than 75% homology over 100 base pairs to the human genome); they are linked to the underlying regions of alignment. The ECRs containing conserved GATA sites are encircled; the proximal ECR constitutes an intron in one (β) of the *Lhx9 *isoforms and is shaded in pink. (**B**) The Nucleotide sequence of the 255 bp fragment (Lhx9_+2xAGATAG) from the internal region of the *Lhx9 *gene (nucleotides +7195/+7450 from the *Lhx9β *translation start site); sequences corresponding to primers are shown in bold; GATA sequences are highlighted in red. Click here for file

## References

[B1] Tevosian SG, Deconinck AE, Cantor AB, Rieff HI, Fujiwara Y, Corfas G, Orkin SH (1999). FOG-2: A novel GATA-family cofactor related to multitype zinc-finger proteins Friend of GATA-1 and U-shaped. Proc Natl Acad Sci U S A.

[B2] Tevosian SG, Deconinck AE, Tanaka M, Schinke M, Litovsky SH, Izumo S, Fujiwara Y, Orkin SH (2000). FOG-2, a cofactor for GATA transcription factors, is essential for heart morphogenesis and development of coronary vessels from epicardium. Cell.

[B3] Novak K (2000). Lost in the FOG. Nat Med.

[B4] Shalaby F, Rossant J, Yamaguchi TP, Breitman ML, Schuh AC (1995). Failure of blood island formation and vasculogenesis in flk-1 deficient mice. Nature.

[B5] Bautch VL, Ambler CA (2004). Assembly and patterning of vertebrate blood vessels. Trends Cardiovasc Med.

[B6] Cantor AB, Orkin SH (2005). Coregulation of GATA factors by the Friend of GATA (FOG) family of multitype zinc finger proteins. Semin Cell Dev Biol.

[B7] Sorrentino RP, Gajewski KM, Schulz RA (2005). GATA factors in Drosophila heart and blood cell development. Semin Cell Dev Biol.

[B8] Crispino JD, Lodish MB, MacKay JP, Orkin SH (1999). Direct association of FOG with GATA-1 is critical for erythroid differentiation and expression of multiple, but not all, GATA-1 target genes. Mol Cell.

[B9] Wang X, Crispino JD, Letting DL, Nakazawa M, Poncz M, Blobel GA (2002). Control of megakaryocyte-specific gene expression by GATA-1 and FOG-1: role of Ets transcription factors. Embo J.

[B10] Tsang AP, Visvader JE, Turner CA, Fujiwara Y, Yu C, Weiss MJ, Crossley M, Orkin SH (1997). FOG, a multitype zinc finger protein, acts as a cofactor for transcription factor GATA-1 in erythroid and megakaryocytic differentiation. Cell.

[B11] Fox AH, Liew C, Holmes M, Kowalski K, Mackay J, Crossley M (1999). Transcriptional cofactors of the FOG family interact with GATA proteins by means of multiple zinc fingers. Embo J.

[B12] Kawabata H, Germain RS, Ikezoe T, Tong X, Green EM, Gombart AF, Koeffler HP (2001). Regulation of expression of murine transferrin receptor 2. Blood.

[B13] Svensson EC, Tufts RL, Polk CE, Leiden JM (1999). Molecular cloning of FOG-2: a modulator of transcription factor GATA-4 in cardiomyocytes. Proc Natl Acad Sci U S A.

[B14] Lu JR, McKinsey TA, Xu H, Wang DZ, Richardson JA, Olson EN (1999). FOG-2, a heart- and brain-enriched cofactor for GATA transcription factors. Mol Cell Biol.

[B15] Crispino JD, Lodish MB, Thurberg BL, Litovsky SH, Collins T, Molkentin JD, Orkin SH (2001). Proper coronary vascular development and heart morphogenesis depend on interaction of GATA-4 with FOG cofactors. Genes Dev.

[B16] Svensson EC, Huggins GS, Lin H, Clendenin C, Jiang F, Tufts R, Dardik FB, Leiden JM (2000). A syndrome of tricuspid atresia in mice with a targeted mutation of the gene encoding Fog-2. Nat Genet.

[B17] Hong W, Nakazawa M, Chen YY, Kori R, Vakoc CR, Rakowski C, Blobel GA (2005). FOG-1 recruits the NuRD repressor complex to mediate transcriptional repression by GATA-1. Embo J.

[B18] Rodriguez P, Bonte E, Krijgsveld J, Kolodziej KE, Guyot B, Heck AJ, Vyas P, de Boer E, Grosveld F, Strouboulis J (2005). GATA-1 forms distinct activating and repressive complexes in erythroid cells. Embo J.

[B19] Deconinck AE, Mead PE, Tevosian SG, Crispino JD, Katz SG, Zon LI, Orkin SH (2000). FOG acts as a repressor of red blood cell development in Xenopus. Development.

[B20] Lin AC, Roche AE, Wilk J, Svensson EC (2004). The N termini of Friend of GATA (FOG) proteins define a novel transcriptional repression motif and a superfamily of transcriptional repressors. J Biol Chem.

[B21] Svensson EC, Huggins GS, Dardik FB, Polk CE, Leiden JM (2000). A functionally conserved N-terminal domain of the friend of GATA-2 (FOG-2) protein represses GATA4-dependent transcription. J Biol Chem.

[B22] Holmes M, Turner J, Fox A, Chisholm O, Crossley M, Chong B (1999). hFOG-2, a novel zinc finger protein, binds the co-repressor mCtBP2 and modulates GATA-mediated activation. J Biol Chem.

[B23] Hobert O, Westphal H (2000). Functions of LIM-homeobox genes. Trends Genet.

[B24] Allan DW, Thor S (2003). Together at last: bHLH and LIM-HD regulators cooperate to specify motor neurons. Neuron.

[B25] Liem KF, Tremml G, Jessell TM (1997). A role for the roof plate and its resident TGFbeta-related proteins in neuronal patterning in the dorsal spinal cord. Cell.

[B26] Bertuzzi S, Porter FD, Pitts A, Kumar M, Agulnick A, Wassif C, Westphal H (1999). Characterization of Lhx9, a novel LIM/homeobox gene expressed by the pioneer neurons in the mouse cerebral cortex. Mech Dev\.

[B27] Retaux S, Rogard M, Bach I, Failli V, Besson MJ (1999). Lhx9: a novel LIM-homeodomain gene expressed in the developing forebrain. J Neurosci.

[B28] Bermingham NA, Hassan BA, Wang VY, Fernandez M, Banfi S, Bellen HJ, Fritzsch B, Zoghbi HY (2001). Proprioceptor pathway development is dependent on Math1. Neuron.

[B29] Birk OS, Casiano DE, Wassif CA, Cogliati T, Zhao L, Zhao Y, Grinberg A, Huang S, Kreidberg JA, Parker KL, Porter FD, Westphal H (2000). The LIM homeobox gene Lhx9 is essential for mouse gonad formation. Nature.

[B30] Failli V, Rogard M, Mattei MG, Vernier P, Retaux S (2000). Lhx9 and Lhx9alpha LIM-homeodomain factors: genomic structure, expression\ patterns, chromosomal localization, and phylogenetic analysis. Genomics.

[B31] Lee KJ, Mendelsohn M, Jessell TM (1998). Neuronal patterning by BMPs: a requirement for GDF7 in the generation of a discrete class of commissural interneurons in the mouse spinal cord. Genes Dev.

[B32] Bendall AJ, Rincon-Limas DE, Botas J, Abate-Shen C (1998). Protein complex formation between Msx1 and Lhx2 homeoproteins is incompatible with DNA binding activity. Differentiation.

[B33] Li DY, Sorensen LK, Brooke BS, Urness LD, Davis EC, Taylor DG, Boak BB, Wendel DP (1999). Defective angiogenesis in mice lacking endoglin. Science.

[B34] Dettman RW, Denetclaw W, Ordahl CP, Bristow J (1998). Common epicardial origin of coronary vascular smooth muscle, perivascular fibroblasts, and intermyocardial fibroblasts in the avian heart. Dev Biol.

[B35] Mikawa T, Fischman DA (1992). Retroviral analysis of cardiac morphogenesis: discontinuous formation of coronary vessels.

[B36] Mikawa T, Gourdie RG (1996). Pericardial mesoderm generates a population of coronary smooth muscle cells migrating into the heart along with ingrowth of the epicardial organ. Dev Biol.

[B37] Perez-Pomares JM, Macias D, Garcia-Garrido L, Munoz-Chapuli R (1998). The origin of the subepicardial mesenchyme in the avian embryo: an immunohistochemical and quail-chick chimera study. Dev Biol.

[B38] Vrancken Peeters MP, Gittenberger-de Groot AC, Mentink MM, Poelmann RE (1999). Smooth muscle cells and fibroblasts of the coronary arteries derive from epithelial-mesenchymal transformation of the epicardium. Anat Embryol (Berl).

[B39] Laugwitz KL, Moretti A, Lam J, Gruber P, Chen Y, Woodard S, Lin LZ, Cai CL, Lu MM, Reth M, Platoshyn O, Yuan JX, Evans S, Chien KR (2005). Postnatal isl1+ cardioblasts enter fully differentiated cardiomyocyte lineages. Nature.

[B40] Komiyama M, Ito K, Shimada Y (1987). Origin and development of the epicardium in the mouse embryo. Anat Embryol (Berl).

[B41] Reese DE, Mikawa T, Bader DM (2002). Development of the coronary vessel system. Circ Res.

[B42] Munoz-Chapuli R, Macias D, Gonzalez-Iriarte M, Carmona R, Atencia G, Perez-Pomares JM (2002). [The epicardium and epicardial-derived cells: multiple functions in cardiac development.]. Rev Esp Cardiol.

[B43] Viragh S, Challice CE (1981). The origin of the epicardium and the embryonic myocardial circulation in the mouse. Anat Rec.

[B44] Hirose T, Karasawa M, Sugitani Y, Fujisawa M, Akimoto K, Ohno S, Noda T (2006). PAR3 is essential for cyst-mediated epicardial development by establishing apical cortical domains. Development\.

[B45] Kolterud A, Wandzioch E, Carlsson L (2004). Lhx2 is expressed in the septum transversum mesenchyme that becomes an integral part of the liver and the formation of these cells is independent of functional Lhx2. Gene Expr Patterns.

[B46] Tevosian SG, Albrecht KH, Crispino JD, Fujiwara Y, Eicher EM, Orkin SH (2002). Gonadal differentiation, sex determination and normal Sry expression in mice require direct interaction between transcription partners GATA4 and FOG2. Development.

[B47] Ovcharenko I, Nobrega MA, Loots GG, Stubbs L (2004). ECR Browser: a tool for visualizing and accessing data from comparisons of multiple vertebrate genomes. Nucleic Acids Res.

[B48] Jurata LW, Pfaff SL, Gill GN (1998). The nuclear LIM domain interactor NLI mediates homo- and heterodimerization of LIM domain transcription factors. J Biol Chem.

[B49] Thaler JP, Lee SK, Jurata LW, Gill GN, Pfaff SL (2002). LIM factor Lhx3 contributes to the specification of motor neuron and interneuron identity through cell-type-specific protein-protein interactions. Cell.

[B50] Glenn DJ, Maurer RA (1999). MRG1 binds to the LIM domain of Lhx2 and may function as a coactivator to stimulate glycoprotein hormone alpha-subunit gene expression. J Biol Chem.

[B51] Sun Y, Liang X, Najafi N, Cass M, Lin L, Cai CL, Chen J, Evans SM (2007). Islet 1 is expressed in distinct cardiovascular lineages, including pacemaker and coronary vascular cells. Dev Biol.

[B52] Murray-Zmijewski F, Lane DP, Bourdon JC (2006). p53/p63/p73 isoforms: an orchestra of isoforms to harmonise cell differentiation and response to stress. Cell Death Differ.

[B53] Qian F, Zhen F, Xu J, Huang M, Li W, Wen Z (2007). Distinct functions for different scl isoforms in zebrafish primitive and definitive hematopoiesis. PLoS Biol.

[B54] Tsuzuki S, Hong D, Gupta R, Matsuo K, Seto M, Enver T (2007). Isoform-specific potentiation of stem and progenitor cell engraftment by AML1/RUNX1. PLoS Med.

[B55] Molle B, Pere S, Failli V, Bach I, Retaux S (2004). Lhx9 and lhx9alpha: differential biochemical properties and effects on neuronal differentiation. DNA Cell Biol.

[B56] Liodis P, Denaxa M, Grigoriou M, Akufo-Addo C, Yanagawa Y, Pachnis V (2007). Lhx6 activity is required for the normal migration and specification of cortical interneuron subtypes. J Neurosci.

[B57] Daubas P, Tajbakhsh S, Hadchouel J, Primig M, Buckingham M (2000). Myf5 is a novel early axonal marker in the mouse brain and is subjected to post-transcriptional regulation in neurons. Development.

[B58] Mukhopadhyay M, Teufel A, Yamashita T, Agulnick AD, Chen L, Downs KM, Schindler A, Grinberg A, Huang SP, Dorward D, Westphal H (2003). Functional ablation of the mouse Ldb1 gene results in severe patterning defects during gastrulation. Development.

[B59] Rincon-Limas DE, Lu CH, Canal I, Botas J (2000). The level of DLDB/CHIP controls the activity of the LIM homeodomain protein apterous: evidence for a functional tetramer complex in vivo. Embo J.

[B60] Bach I (2000). The LIM domain: regulation by association. Mech Dev.

[B61] Ostendorff HP, Peirano RI, Peters MA, Schluter A, Bossenz M, Scheffner M, Bach I (2002). Ubiquitination-dependent cofactor exchange on LIM homeodomain transcription factors. Nature.

[B62] Hiratani I, Yamamoto N, Mochizuki T, Ohmori SY, Taira M (2003). Selective degradation of excess Ldb1 by Rnf12/RLIM confers proper Ldb1 expression levels and Xlim-1/Ldb1 stoichiometry in Xenopus organizer functions. Development.

[B63] Manuylov NL, Fujiwara Y, Poulat F, Tevosian SG, Adameyko (2007). The regulation of Sox9 gene expression by the GATA4/FOG2 transcriptional complex in dominant XX sex reversal mouse models. Dev Biol.

[B64] Manuylov NL, Smagulova FO, Tevosian SG (2007). Fog2 excision in mice leads to premature mammary gland involution and reduced Esr1 gene expression. Oncogene.

[B65] Wilkinson DG (1992). In Situ Hybridization.

[B66] Mudry RE, Houston-Cummings NR, Veselov AP, Gregorio CC, Tevosian SG, Adameyko (2005). Expression and regulation of mouse SERDIN1, a highly conserved cardiac-specific leucine-rich repeat protein. Dev Dyn.

[B67] Sambrook J, Fritsch EF, Maniatis T (1989). Molecular cloning: a laboratory manual.

