# Supplementary material for: GATA4/FOG2 transcriptional complex regulates Lhx9 gene expression in murine heart development
Source: BMC Dev Biol. 2008 Jun 24;8:67. doi: 10.1186/1471-213X-8-67 (PMC2447832; doi:10.1186/1471-213X-8-67)
Supplement: Additional file 1 — Differentially Expressed Genes in E13.5 Control vs. Mutant Hearts [file 1471-213X-8-67-S1.pdf]

Supplemental Table 1.
Differentially Expressed Genes in E13.5 Control vs. *Fog2*$^{-/-}$ hearts
(cut-off point ~2.5 fold)

Gene order	Affymetrix Probe	Fold Change	Sequence Accession Number	Gene Symbol	Gene Name
**Downregulated genes**					
1.	1419606_a_at	**-4,21**	NM_011618.1	Tnnt1	troponin T1
2.	1451203_at	**-4,03**	BC025172.1	Mb	myoglobin
3.	1448545_at	**-3,88**	AU021035	Sdc2	syndecan 2
4.	1433184_at	**-3.22**	AK020162.1	Cacna1c	calcium channel, voltage-dependent, L type, alpha 1C subunit
5.	1417023_a_at	**-3,53**	NM_024406.1	Fabp4	fatty acid binding protein 4
6.	1419012_at	**-3.24***	AF107306.1	Zfpm2	Zinc finger protein, multitype 2
7.	1438072_at	**-2,7**	BB242844	Nfib	nuclear factor I/B
8.	1447231_at	**-2,68**	BM123508	Slc8a1	solute carrier family 8 (sodium/calcium exchanger
9.	1441624_at	**-2,66**	BB479893	Sorbs2	sorbin and SH3 domain containing 2
10.	1446921_at	**-2,66**	BM204670	Grb10	growth factor receptor bound protein 10
11.	1458081_at	**-2,59**	BB477614	NA	hypothetical protein
12.	1457797_at	**-2,52**	AV340788	Slc8a1	solute carrier family 8 (sodium/calcium exchanger), member 1
13.	1417614_at	**-2,44**	NM_007710.1	Ckm	creatine kinase, muscle
14.	1438651_a_at	**-2,41**	BB483357	Agtrl1	angiotensin receptor-like 1
15.	1438878_at	**-2,41**	AU067682	6430537K16Rik	6430537K16Rik
**Upregulated genes**					
1.	1416645_a_at	**10.08**	NM_007423.1	Afp	alpha fetoprotein
2.	1454866_s_at	**7.71**	BQ176424	Clic6	chloride intracellular channel 6
3.	1427119_at	**4.39**	AV066321	Spink4	serine protease inhibitor, Kazal type 4
4.	1427126_at	**3.36**	M12573.1	Hspa1b	heat shock protein 1B
5.	1448949_at	**3.27**	NM_007607.1	Car4	carbonic anhydrase 4
6.	1416103_at	**3.16**	BF608615	Ywhaz	tyrosine 3-monooxygenase/tryptophan 5-monooxygenase activation protein, zeta polypeptide
**7.**	**1419324_at**	**3.15**	**NM_010714.1**	**Lhx9**	**LIM homeobox protein 9**
8.	1423691_x_at	**3.12**	M21836.1	Krt2-8	keratin complex 2, basic, gene 8
9.	1436043_at	**3.07**	BB508825	Scn7a	sodium channel, voltage-gated, type VI, alpha polypeptide
10.	1452388_at	**3.05**	AW763765	Hspa1a	heat shock protein 1A
11.	1433930_at	**3.02**	BG094050	Hpse	heparanase
12.	1439622_at	**2.88**	AV291679	Rassf4	Ras association (RalGDS/AF-6) domain family 4
13.	1418934_at	**2.85**	NM_011839.1	Mab21l2	mab-21-like 2 (C. elegans)
14.	1448756_at	**2.69**	NM_009114.1	S100a9	S100 calcium binding protein A9 (calgranulin B)
15.	1435761_at	**2.68**	AW146083	Stfa1	stefin A1
16.	1428547_at	**2.57**	AV273591	Nt5e	5' nucleotidase, ecto
17.	1416468_at	**2.56**	NM_013467.1	Aldh1a1	aldehyde dehydrogenase family 1, subfamily A1
18.	1430700_a_at	**2.55**	AK005158.1	Pla2g7	phospholipase A2, group VII (platelet-activating factor acetylhydrolase, plasma)
19.	1448169_at	**2.53**	NM_010664.1	Krt1-18	keratin complex 1, acidic, gene 18

* Calculated by the Gene traffic program using the combined value for **both** tags corresponding to *Zfpm2* (*Fog2*). The call value for the tag corresponding to the deleted region (encoding zinc finger regions; Tevosian et al., 2000) of *Zfpm2* (*Fog2*) is absent (A) in the *Fog2*$^{-/-}$ sample.
