# Supplementary figures and images for: GATA4/FOG2 transcriptional complex regulates Lhx9 gene expression in murine heart development
Source: BMC Dev Biol. 2008 Jun 24;8:67. doi: 10.1186/1471-213X-8-67 (PMC2447832; doi:10.1186/1471-213X-8-67)

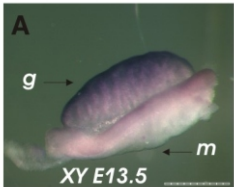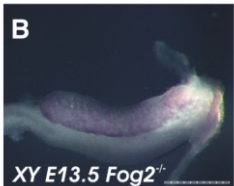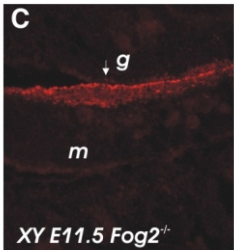

Supplement: Additional file 2 — (A-B) An E13.5 XY gonad from the control (A) and Fog2-/- mutant (B) embryos were examined by in situ hybridization with an Lhx9α/β RNA probe. The mutant sample lacks the sex cords resulting from a block in male sexual development [46]; g-gonad, m-mesonephros. (C) A specific staining in the genital ridge of the E11.5 Fog2-/- embryo with LHX2/9 antibody [31]. [file 1471-213X-8-67-S2.pdf]
