# Supplementary material for: GATA4/FOG2 transcriptional complex regulates Lhx9 gene expression in murine heart development
Source: BMC Dev Biol. 2008 Jun 24;8:67. doi: 10.1186/1471-213X-8-67 (PMC2447832; doi:10.1186/1471-213X-8-67)
Supplement: Additional file 3 — (A) In silico identification of the putative GATA elements in the Lhx9 gene locus. An ECR browser output showing the conservation profiles of the human region in comparison with the monkey, mouse, rat, dog and opossum genomes. Exons are shown in blue and yellow; the blue bars correspond to the protein coding regions while yellow bars depict the UTRs. The 5'-3' orientation of the gene is shown by the blue arrow lines on top. Dark red bars show the distribution of ECRs (the areas of the respective genome with greater than 75% homology over 100 base pairs to the human genome); they are linked to the underlying regions of alignment. The ECRs containing conserved GATA sites are encircled; the proximal ECR constitutes an intron in one (β) of the Lhx9 isoforms and is shaded in pink. (B) The Nucleotide sequence of the 255 bp fragment (Lhx9_+2xAGATAG) from the internal region of the Lhx9 gene (nucleotides +7195/+7450 from the Lhx9β translation start site); sequences corresponding to primers are shown in bold; GATA sequences are highlighted in red. [file 1471-213X-8-67-S3.pdf]

A.

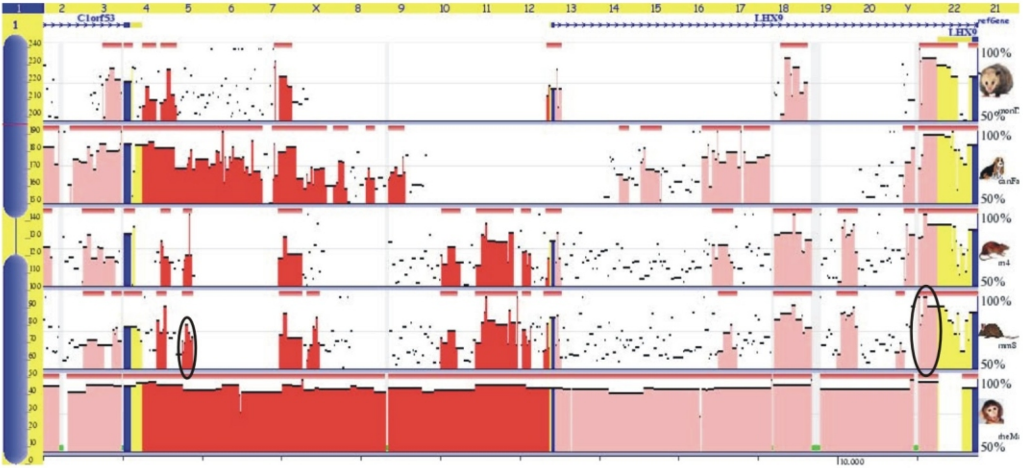

http://ecrbrowser.dcode.org/browser.php?jID=18581160&location=chr1:196143837-196151677&action=Out1.5x

B.

Lhx9_+2xAGATAG

AGGTACTATCTGCTGGCCGTAGACAAACAGTGGCATCTTAGGTGCCTGAA
GTGCTGTGAATGTAAGCTGGCTCTGGAATCTGAGCTCACCTGCTTTGCCA
AGGACGGTAGCATTTACTGCAAGGAGGATTATTACAGTACTGCCCCCACC
CCCAGCCCCTTCACACACTCCTCTGACCCAATTCACCAGATAGAACCACT
GCGATAGAGCCACTGCGAG**AATCCGGAGTCCCTTCTCTGTCCCTT**
